# T-cell receptor and B-cell receptor repertoires profiling in pleural tuberculosis

**DOI:** 10.3389/fimmu.2024.1473486

**Published:** 2024-11-27

**Authors:** Fengjiao Du, Yunyun Deng, Ling Deng, Boping Du, Aiying Xing, Hong Tao, Hua Li, Li Xie, Xinyong Zhang, Tao Sun, Hao Li

**Affiliations:** ^1^ National Clinical Laboratory on Tuberculosis, Beijing Tuberculosis and Thoracic Tumor Institute, Beijing Chest Hospital, Capital Medical University, Beijing, China; ^2^ Hangzhou ImmuQuad Biotechnologies, Hangzhou, China; ^3^ Institute of Wenzhou, Zhejiang University, Wenzhou, China; ^4^ Center for Infectious Disease Research, School of Medicine, Tsinghua University, Beijing, China; ^5^ College of Veterinary Medicine, China Agricultural University, Beijing, China

**Keywords:** pleural tuberculosis, T cell receptor, B cell receptor, deep sequencing, antibody

## Abstract

**Background:**

Tuberculosis (TB) is a leading cause of death worldwide from a single infectious agent. In China the most common extra-pulmonary TB (EPTB) is pleural tuberculosis (PLTB). An important clinical feature of PLTB is that the lymphocytes associated with TB will accumulate in the pleural fluid. The adaptive immune repertoires play important roles in *Mycobacterium tuberculosis* (Mtb) infection.

**Methods:**

In this study, 10 PLTB patients were enrolled, and their Peripheral Blood Mononuclear Cells(PBMCs) and Pleural Effusion Mononuclear Cells(PEMCs) were collected. After T cells were purified from PBMCs and PEMCs, high-throughput immunosequencing of the TCRβ chain (TRB), TCRγ chain(TRG), and B cell receptor(BCR) immunoglobulin heavy chain (IGH) were conducted on these samples.

**Results:**

The TRB, TRG, and BCR IGH repertoires were characterized between the pleural effusion and blood in PLTB patients, and the shared clones were analyzed and collected. The binding activity of antibodies in plasma and pleural effusion to Mtb antigens was tested which indicates that different antibodies responses to Mtb antigens in plasma and pleural effusion in PLTB patients. Moreover, GLIPH2 was used to identify the specificity groups of TRB clusters and Mtb-specific TRB sequences were analyzed and collected by VJ mapping.

**Conclusion:**

We characterize the adaptive immune repertoires and identify the shared clones and Mtb-specific clones in pleural effusion and blood in PLTB patients which can give important clues for TB diagnosis, treatment, and vaccine development.

## Introduction

Tuberculosis (TB) is one of the leading causes of death from a single infectious agent worldwide, ranking above HIV/AIDS (1). TB mortality has been severely impacted by the COVID-19 pandemic in the 3 years 2020–2022. There are an estimated 1.30 million deaths and 10.6 million people falling ill with TB in 2022 (1). The only licensed tuberculosis vaccine is *Bacillus Calmette-Guerin* (BCG), which has shown variable efficacy and provides partial protection against TB in children (2). Therefore, there is an urgent need to develop a better TB vaccine.

Although *Mycobacterium tuberculosis* (Mtb) usually infects the lung and causes pulmonary tuberculosis, approximately 25% of patients initially have extra-pulmonary TB (EPTB) presentation mostly in the pleura and lymph nodes (3). In China, the most common EPTB is pleural tuberculosis (PLTB), which accounts for 50.15% (4). There is still a great challenge ahead for PLTB diagnosis and treatment because of the paucibacillary mycobacterial infection and the emergence of drug-resistant strains (3–5). CD4+ T cells have important protective roles in controlling the initiation and progression of PLTB. The cytokine interferon-gamma (IFN-γ) and interleukin-12 (IL-12) level in pleural effusion is significantly higher than in the peripheral blood (6–8). Additionally, the other types of T cells such as CD8+, γδ T, and Th17 cells also play important roles in resisting Mtb infection in pleural effusion (9, 10). Emerging evidence has shown that humoral responses have protection against Mtb infection (11–17); however, there are few studies on the roles of B cells in PLTB. It is worth noting that some of the PLTB patients can recover without chemotherapy treatments (18, 19), which gives us suggestions that there may be protective immune responses against Mtb in PLTB patients and need to be further studied.

T cells can recognize different pathogens by the T-cell receptors (TCRs) on the surface, which is mainly because of the diversity of the hyper-variable diversity of amino acids sequence of the complementarity-determining region 3 (CDR3) of TCR. Human T-cell receptors are formed as an αβ or γδ heterodimer, and in 95% of T cells, the TCR consists of an α and a β chain, whereas only in 5% of T cells, the TCR consists of γ and δ chains. TCRα and TCRγ genes are assembled via recombination of variable segments (V) and joining gene segments (J), TCR β and TCRδ genes via the recombination of variable (V), diversity (D), and joining (J) segments (20). During thymic selection, more than 1×10^13^ possible T-cell receptors can be selected, and TCRs have three complementary determining regions (CDR1, CDR2, and CDR3). The CDR3 region is the most important determinant of T-cell antigen specificity and mediates T-cell diversity, which can help the host to fight against different pathogens via the immune responses (21). The specific CDR3 sequence frequency can reflect the expansion of the corresponding T-cell clones. The CDR3 length distribution analysis has been performed to evaluate the TCR αβ and TCR γδ repertoires under different physiologic conditions and pathological situations (22–25). The diversity of TCR repertoires is closely related to the pathogens’ infection and disease progression such as HIV, EBV, and influenza A virus (26–29). After Mtb enters the body, B cells and T cells can work together to prevent infection. T helper cells can help B cells produce high-affinity antibodies and become memory B cells, and B cells can modulate T-cell immune response by different mechanisms such as antigen presentation and antibody and cytokine production (30–32). The previous studies indicate that the antibodies produced in pleural effusion can recognize Mtb antigens, and the SDF-1/CXCR4 axis may facilitate the circulating B cells into the tuberculous pleural space (33–36). Like T cells, B cells have receptors called BCR on their surface. BCRs possess a highly diverse pair of variable heavy (V_H_) and variable light (V_L_) chains, and somatic recombination of V-, D- and J-gene germline segments and somatic hyper-mutation results in an estimated human BCR diversity of 10^13^ (37). The diversity of the BCR repertoires has important roles in protection against different pathogens (37, 38).

High-throughput sequencing (HTS) is an innovative and advanced technology for the researcher studying adaptive immunity by analysis of the T cells and B cells’ repertoires and can generate large datasets for revealing insights on TCR/BCR clonal selection, expansion, and evolution (39–42). Additionally, deep sequencing is also widely used in studying the immune responses following infection or vaccination (43, 44). The main feature of PLTB is that the lymphocytes are prone to be enriched in the pleural effusion of PLTB patients, and the bacteria or the antigens can be detected in the pleural effusion (3, 8). Therefore, the pleural cavity provides a relatively closed environment, which can facilitate the interaction of Mtb and the lymphocytes in the pleural effusion and a good TB research model. In this study, we use high-throughput deep sequencing to analyze and characterize the TCRβ, TCRγ, and BCR IGH repertoires, which can give us an in-depth understanding of immune responses in PLTB patients, as well as make suggestions on the TB diagnosis and vaccine development.

## Methods

### Patients samples

A total of 10 cases of pleural tuberculosis (PLTB) were enrolled in this study from September 2018 to November 2019 in Beijing Chest Hospital (Beijing, China). All the patients had definitive TPE diagnosis by China WS 288-2017 Tuberculosis Diagnosis Standard (1): Clinical samples are tested *Mycobacterium tuberculosis* positive by bacteriology or molecular biology methods (2). Pleural biopsy specimens demonstrate tuberculosis granuloma or caseous necrosis by pathological examination. All the patients had no HIV infection, hepatitis virus infection, diabetes, or autoimmune diseases and did not have the TB treatments and immune-modulating drugs. Medical information, human whole blood, and pleural fluid samples were obtained from the patients after they signed the written consent form. This study was approved by the ethics committee of Beijing Chest Hospital (No. BJXK-2015-08).

### Peripheral blood mononuclear cell and pleural effusion mononuclear cell preparation

Heparinized peripheral blood and pleural effusion samples were collected and processed within 2 h of collection. Peripheral blood mononuclear cells (PBMCs) were isolated from 6 ml of whole blood by Ficoll-Paque (GE Healthcare, Germany) gradient centrifugation (12). A 40-ml pleural effusion was centrifuged at 500g for 10 min and then the supernatants were discarded, the sediments were suspended in 5 ml RPMI 1640, and then pleural effusion mononuclear cells (PEMCs) were isolated by the Ficoll-Paque (GE Healthcare, Germany) gradient centrifugation method as above.

### Immunomagnetic isolation of T cells

The T cells were isolated from the collected PBMCs and PEMCs by Pan T Cell Isolation Kit (Miltenyi Biotech) according to the manufacturer’s instructions. Briefly speaking, PBMCs and PEMCs were centrifuged at 300g for 10 min, the supernatant was aspirated, and 40 µl 0.5% FBS/PBS per 10^7^ cells was used for suspension. 10 µl of Pan T Cell Biotin-Antibody cocktail per 10^7^ cells was added, mixed well, and incubated for 10 min at 4°C in the refrigerator. 30 µl of 0.5% FBS/PBS and 20 µl of Pan T Cell Microbead cocktail per 10^7^ cells were added, mixed well, and incubated for an additional 10 min at 4°C in the refrigerator. An LS column was placed in the magnetic field of a suitable MACS separator, and 3 ml of 0.5% FBS/PBS was used to wash the LS column. Cell suspension was applied onto the column. Flow-through containing unlabeled cells, representing the enriched T-cell fraction, was collected. The column was washed with 3 ml of buffer. Unlabeled cells that pass through, representing the enriched T cells, were collected and combined with the flow-through above. The column was removed from the separator and placed on a 15-ml falcon tube. 5 ml of buffer was pipetted onto the column, and the magnetically labeled non-T cells (containing B cells) were immediately flushed out by firmly pushing the plunger into the column. The cells will be used for DNA extraction for B-cell repertoire deep sequencing. The purity of isolated T cells was identified by anti-CD3-FITC antibodies by flow cytometry analysis. Briefly speaking, 5 µl anti-CD3-FITC antibodies was added into the sample tubes and incubated at 4° for 1 h in the refrigerator. The samples were washed with 0.5% FBS/PBS three times, 5% FBS/PBS was used to suspend the cells, and a BD C6 FACS machine was used to analyze the samples. Data were analyzed using FlowJo software.

### Genome DNA preparation

The T-cell DNA from PBMCs or PEMCs was extracted by QIAamp Blood Mini Kits (Qiagen, Germany) according to the manufacturer’s instructions. The left cells of PBMCs and PEMCs after T-cell isolation were used for DNA extraction for B-cell deep sequencing, and the DNA was also extracted as above. The DNA samples need to pass quality control (concentration ≥70 ng/μl; 1.7 ≤OD260/280 ≤1.9) and then be used for deep sequencing.

### TRB, TRG, and IGH deep sequencing

DNA samples were analyzed by High-Throughput Sequencing (HTS) of TRB and TRG using the ImmuHub^®^ TCR profiling system at a deep level (ImmuQuad Biotech, Hangzhou, China). Briefly, a multiplex PCR amplification protocol was used. Briefly, PCR (PCR-1) amplification was carried out by use of 1 μg DNA with 31 TRBV, 12 TRBJ primers for TRB and 6 TRGV, 3 TRGJ primers for TRG and 16 IGHV, 2 IGHJ primers for IGH by the Multiplex PCR Kit (QIAGEN, Germany) to amplify the third complementarity-determining region (CDR3) of TCRB, TCRD, and IGH.

The amplified TRB, TRG, and IGH products were purified with the Agencourt AMPure XP beads (A63882, Beckman Coulter, Inc.). To prepare final libraries compatible with the Illumina^®^ sequencing platform, PCR (PCR-2) was applied and the Illumina^®^ sequencing indices were added. Final PCR products were purified by the Agencourt AMPure XP beads (A63882, Beckman Coulter, Inc.) again. Purified final PCR products were then analyzed by the Agilent 2100 Bioanalyzer System (Agilent) to determine the molar concentration for dilution required for sequencing samples pool preparation. Sequencing was performed on an Illumina NovaSeq^®^ system with PE150 mode (Illumina).

### Antibodies titer determination by ELISA

The Nunc MaxiSorp high-binding 96-well ELISA plates were used for all ELISA experiments. For plasma or pleural effusion binding to antigens, plates were coated overnight at 4°C with 1 μg/well of PstS1 and purified LAM. For plasma or pleural effusion binding to Mtb strains, plates were coated overnight at 4°C with 1 μg/well H37Rv lysates or 1×10^7^ CFU/well heat-killed H37Rv bacteria. The plates were washed three times with 1× PBST and blocked with 200 μl of 2% BSA solution in PBST for 1.5 h at 37°C. The plates were washed three times with PBST, and then 1:100 diluted plasma and pleural effusion supernatant were added for 100 μl/well, respectively, and incubated for 2 h at 37°C. The plates were washed four times with PBST and then 1:10,000 diluted HRP-conjugated Goat Anti-Human IgG/IgM/IgA H&L (Abcam) was added and incubated at 37°C for 1 h. Aspirated wells were washed four times with PBST, and 100 μl TMB was added into each well for 10 min–15 min at room temperature and then 50 μl of 1 M H_3_PO_4_ into the wells to stop the reaction. The plates were read at 450 nm by Multiscan™ GO Microplate Spectrophotometer (Thermo Fisher), and the collected data were analyzed by Prism 8 software.

### Deep sequencing data analysis

The raw sequencing data of TRB, TRG, and IGH were then aligned with the IMGT^®^ VDJ database with IgBLAST (NCBI) and PCR amplification and sequencing error correction based on clone frequency. The resulting nucleotide and AA sequences of TRB, TRG, and IGH CDR3 were determined, and those with out-of-frame and stop codon sequences were removed from the identified repertoires. We further defined amounts of each TRB, TRG, and BCR IGH clonotype by adding numbers of TRB, TRG, and BCR IGH clones sharing the same nucleotide sequence of CDR3. The Morisita index was analyzed to assess the similarities of TRB, TRG, and IGH repertoires separately between the pleural effusion and blood in PLTB patients. The Morisita’s overlap index is from 0 to 1, in which 0 is no similarity and 1 is fully matched (42, 45, 46). The clonality index and Shannon’s index of diversity were calculated and compared between different groups (47). The TRB, TRG, and BCR IGH analysis algorithm and plotting were performed with R (version 3.5.1).

### High-resolution HLA typing by NGS

The HLA locus-specific sequences (HLA-A, HLA-B, HLA-C, HLA-DRB1, HLA-DQB1, and HLA-DPB1) were amplified by the NGSgo^®^-AmpX kit (GenDx, Utrecht, Netherlands), and the amplicons were pooled. Run gel electrophoresis to check the amplification quality. Fragmentation and end-repair were performed by the NGSgo^®^-LibrX kit (GenDx). Fragments were ligated to the barcode-labeled X adapter by GENDX NGSgo^®^-IndX (GenDx). Fragments with an average size of 200 bp were selected using AMPure XP Beads (Beckman Coulter, CA). Libraries were pooled in equimolar concentrations, and the final library concentration was measured using the NEB Next Library Quant Kit (NEB). The HLA libraries were sequenced on the Illumina MiSeq, using a standard flow cell. The NGS data were analyzed using the HLA typing software package NGSengine 2.0.0.5095 software (GenDx).

### TCR specificity group identification using GLIPH2

The specificity groups of TCRB clusters that were predicted to share the same antigen specificity were analyzed by GLIPH2 (grouping of lymphocyte interactions with paratope hotspots two algorithms) based on TRB sequence similarity (48). The GLIPH2 algorithms cluster the enriched TCRs targeting restricted sets of epitopes by analyzing common motifs in the RAW dataset compared with the reference dataset. The algorithm GLIPH2, reference CDR3β sequences, and tutorial are available from the following link: http://50.255.35.37:8080 (48).

### Mtb-specific TCR analysis

The TRB sequences of the pleural effusion and blood were submitted to VDJDB and the McPAS-TCR database to do the VJ mapping. The Mtb-specific TRB sequences were collected and used for the shared TCR clones identification by Venn analysis. The TRB sequences for 10 healthy controls (HCs) were obtained from the Adaptive Biotechnologies immuneACCESS (https://clients.adaptivebiotech.com/pub/tcrbv4-control) (49).

### Statistical analysis

Tabulated data were analyzed in GraphPad PRISM 8 (GraphPad Software Inc). Comparisons between groups were performed using two-tailed T-tests or ANOVA Kruskal–Wallis test. *p <0.05, **p <0.01, and ***p <0.001 were considered statistically significant.

## Results

### Study design and TCR/BCR deep sequencing of PLTB patient lymphocytes

**Figure 1:**
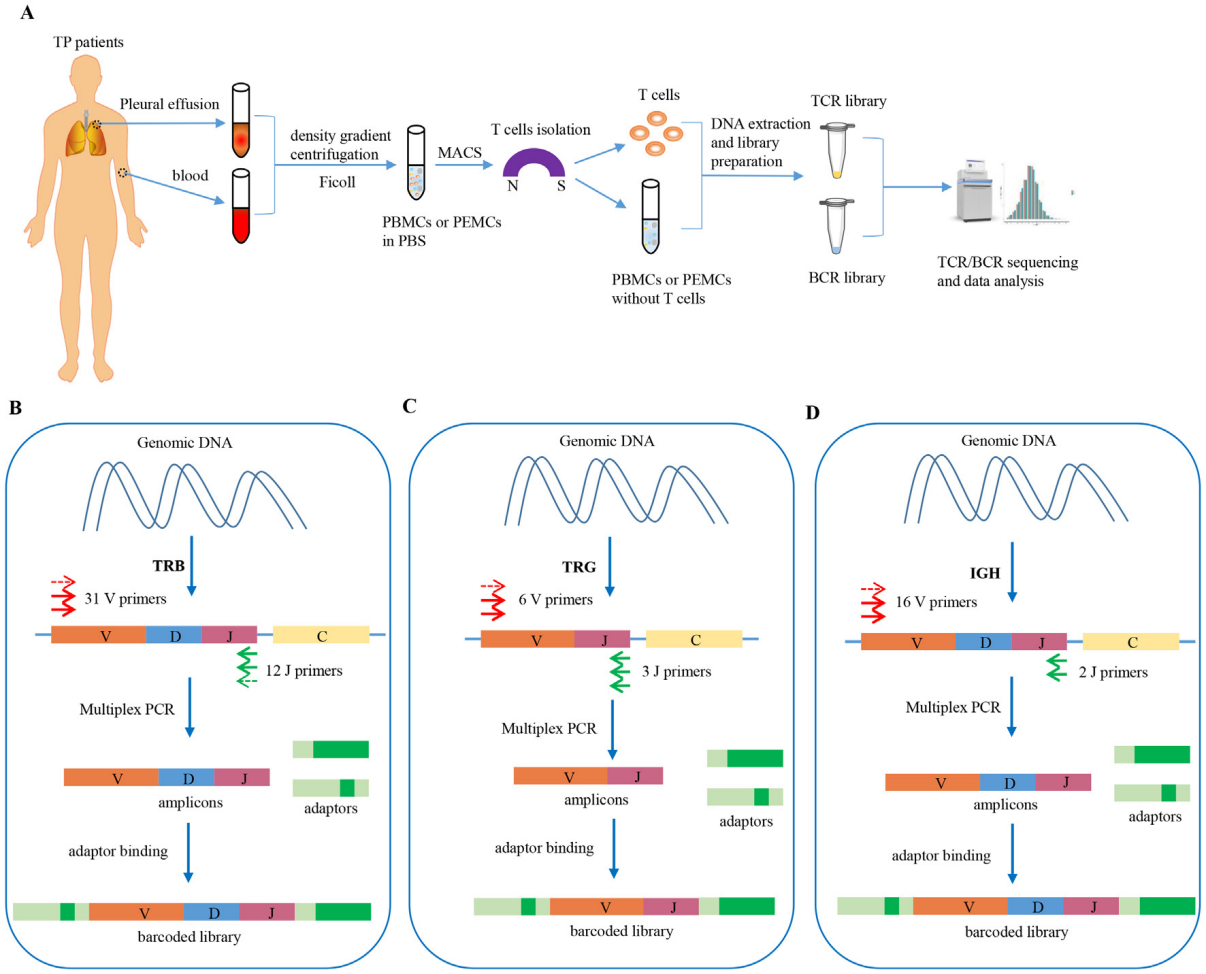
Schematic workflow of TCR/BCR repertoires deep sequencing in PLTB patients and workflow of TRB, TRG, and BCR IGH library preparation. **(A)** Heparinized peripheral blood and pleural effusion samples were collected from the enrolled patients, and the PBMCs and PEMCs were isolated by the Ficoll-Paque gradient centrifugation method. The T cells were isolated from the PBMCs and PEMCs by Pan T Cell Isolation. The DNA of the purified T cells was extracted for TRB/TRG deep sequencing, and the left cells of PBMCs or PEMCs after T-cell isolation were used for DNA extraction for B-cell deep sequencing. Sequencing was performed on an Illumina NovaSeq^®^ system with PE150 mode (Illumina). **(B)** TRB library preparation procedures using Multiplex PCR. **(C)** TRG library preparation procedures using Multiplex PCR. **(D)** IGH library preparation procedures using Multiplex PCR.

We performed TCR/BCR deep sequencing of lymphocytes from PLTB patients. In this study, we collected 10 pleural tuberculosis patients (**Table 1**). All the patients have the PLTB clinical symptoms and are confirmed as PLTB by Mtb culture, pleura GeneXpert, or histopathologic examination (**Table 1**). Patients 102 and 108 also have pulmonary tuberculosis. The pleural effusions and peripheral blood were collected from the PLTB patients and subjected to density gradient separation for isolation of PEMCs and PBMCs. T cells from each patient were purified from PEMCs and PBMCs using negative immune-magnetic enrichment. The isolated T cells were stained by the FITC staining anti-CD3 antibody and then analyzed by flow cytometry. The results indicate that the purity of the isolated T cells from both the blood and pleural effusion is more than 90%, which is good enough to do T-cell receptor deep sequencing (**Supplementary Figure S1**). The T cells’ DNA was extracted, and libraries were prepared for performing TRB and TRG deep sequencing on an Illumina NovaSeq^®^ system. To maximize the utilization of the precious samples, the left cells of PBMCs and PEMCs after T-cell isolation were extracted DNA for BCR IGH deep sequencing (**Figure 1A**). For the TRB, TRG, and IGH library preparation (**Figures 1B–D**), the genomic DNA was used as the template to amplify the CDR3 of TRB, TRG, and IGH by multiplex PCR. The second round of PCR was used to add the sequencing indices (adapters) to CDR3 PCR products to get the barcoded libraries. The prepared libraries were performed on an Illumina NovaSeq^®^ system with PE150 mode (Illumina).

**Table 1:** Clinical characteristics of PLTB patients enrolled in this study.

Patient	Age yrs	Gender	Clinical diagnosis	Complications	Mtb culture	Histopathologic Examination	PE X-pert	Sequencing
E10	20	female	pleural TB	No	Negative	Negative	Positive	TCR
E15	58	male	pleural TB	No	Negative	Negative	Positive	TCR/BCR
E19	56	male	pleural TB	No	Negative	Negative	Positive	TCR/BCR
E22	45	male	pleural TB	No	Negative	Positive	Positive	TCR
E96	41	female	pleural TB	No	Positive	Negative	Positive	BCR
E99	35	male	pleural TB	No	Positive	Negative	Negative	TCR/BCR
E100	68	male	pleural TB	No	Negative	Negative	Positive	TCR/BCR
E101	15	female	pleural TB	No	Negative	Negative	Positive	TCR/BCR
E102	29	female	pleural TB+TB	No	Negative	Negative	Positive	TCR/BCR
E108	30	female	pleural TB+TB	No	Positive	Negative	Negative	TCR/BCR

### Characterization of TRB repertoires in PLTB patients

**Figure 2:**
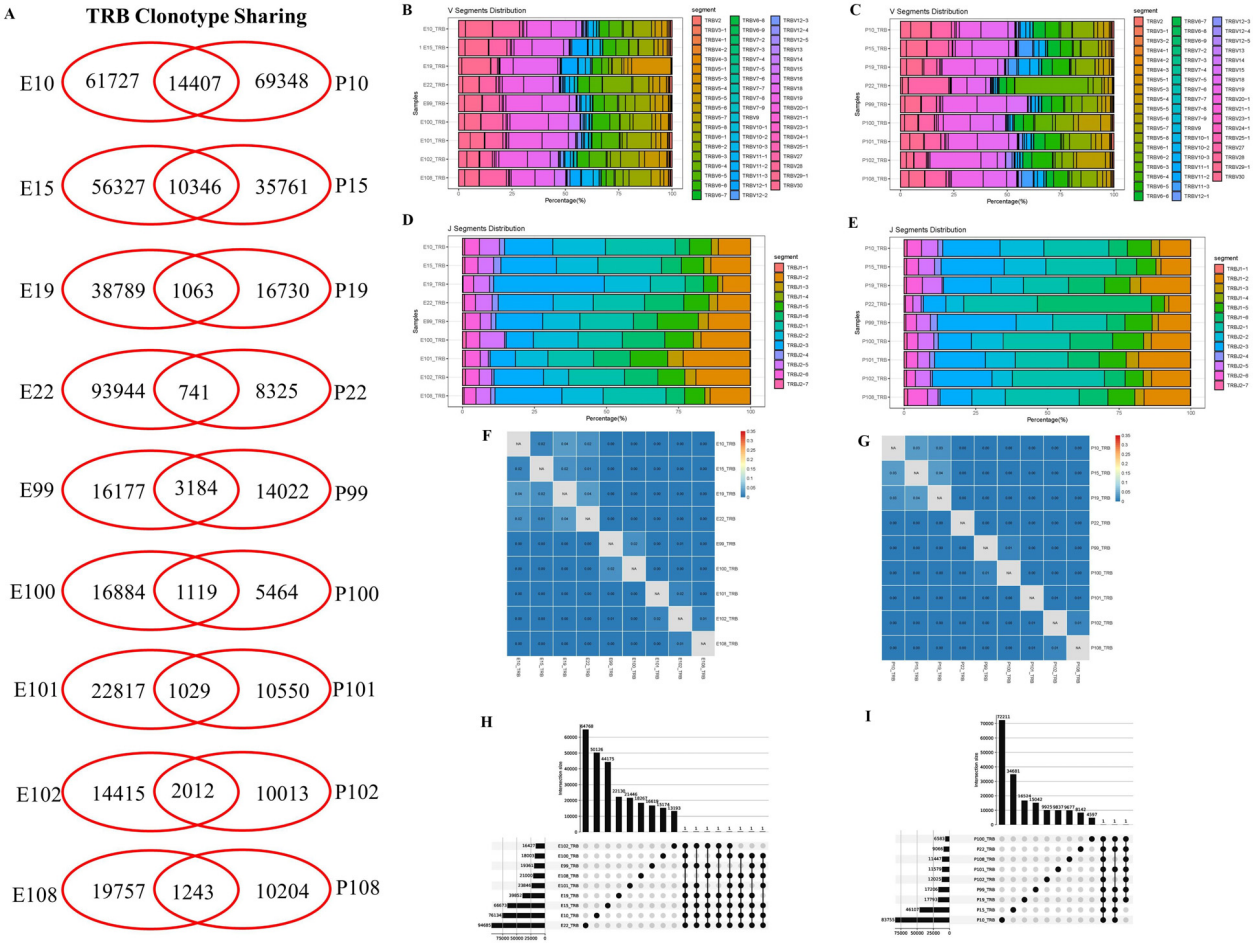
Characterization of T-cell receptor β repertoires between the pleural effusion and blood in PLTB patients. **(A)** TCR sharing across the pleural effusion and blood in PLTB patients. Venn diagram shows the sharing of TRB clonotypes across the PE and blood. Values indicate the number of distinct or shared clonotypes. **(B, C)** The distribution of TRB V gene segment usage in the pleural effusion **(B)** and blood **(C)**. **(D, E)** The TRB J gene segments usage in the pleural effusion **(D)** and blood **(E)**. **(F, G)** Morisita index was used to assess the similarity of TRB repertoires between the pleural effusion **(F)** and blood **(G)** in PLTB patients. **(H, I)** Multi-Venn analysis of the same TRB clonotypes found in pleural effusion **(H)** or blood samples **(I)** in PLTB patients. The numbers shown on the left of the yellow bars mean the total clonotypes in each patient. The dots and connection lines mean the samples analyzed individually or across the selected samples, and the numbers on top of the black bars mean the unique clonotypes in each sample and the public clonotypes across the selected compared samples.

The clonal diversities are analyzed and compared between the pleural effusion and blood in PLTB patients. According to the results, each patient has the shared TRB clones in the pleural effusion and blood by Venn analysis, and there are no differences of clonality and Shannon index between the pleural effusion or blood in PLTB patients (**Figure 2A**; **Supplementary Figure S3**). These give us suggestions that at the early stage of Mtb infection, T cells can enter the pleural effusion from the peripheral blood in PLTB patients. Although there are similar patterns of TRB V or J segment distributions in the pleural effusion and blood, there are also some differences in V or J gene usage frequency. For example, the TRBV5-3 and TRBJ2-3 segments both have significantly higher usage proportions in the pleural effusion compared with the blood in patient 19 (**Figures 2B–E**). Morisita index analysis indicates that there are low similarities of TRB repertoires between the pleural effusion and blood in PLTB patients (**Figures 2F, G**). Moreover, the TRB CDR3 length, clonality frequencies, and VJ combination usage in the pleural effusion and blood of each PLTB patient were analyzed separately (**Supplementary PPT Data File1**-**3**). To further identify the specific Mtb TCRB clones, we analyzed the clones that appeared in at least seven pleural effusions or blood of the nine PLTB patients. The results indicate that there are three clones shared by eight pleural effusions, one shared by nine blood samples (**Figures 2H, I**; **Supplementary Excel**). Whether these clones are Mtb-specific or protective clones needs to be further researched.

### Characterization of TRG repertoires in PLTB patients

**Figure 3:**
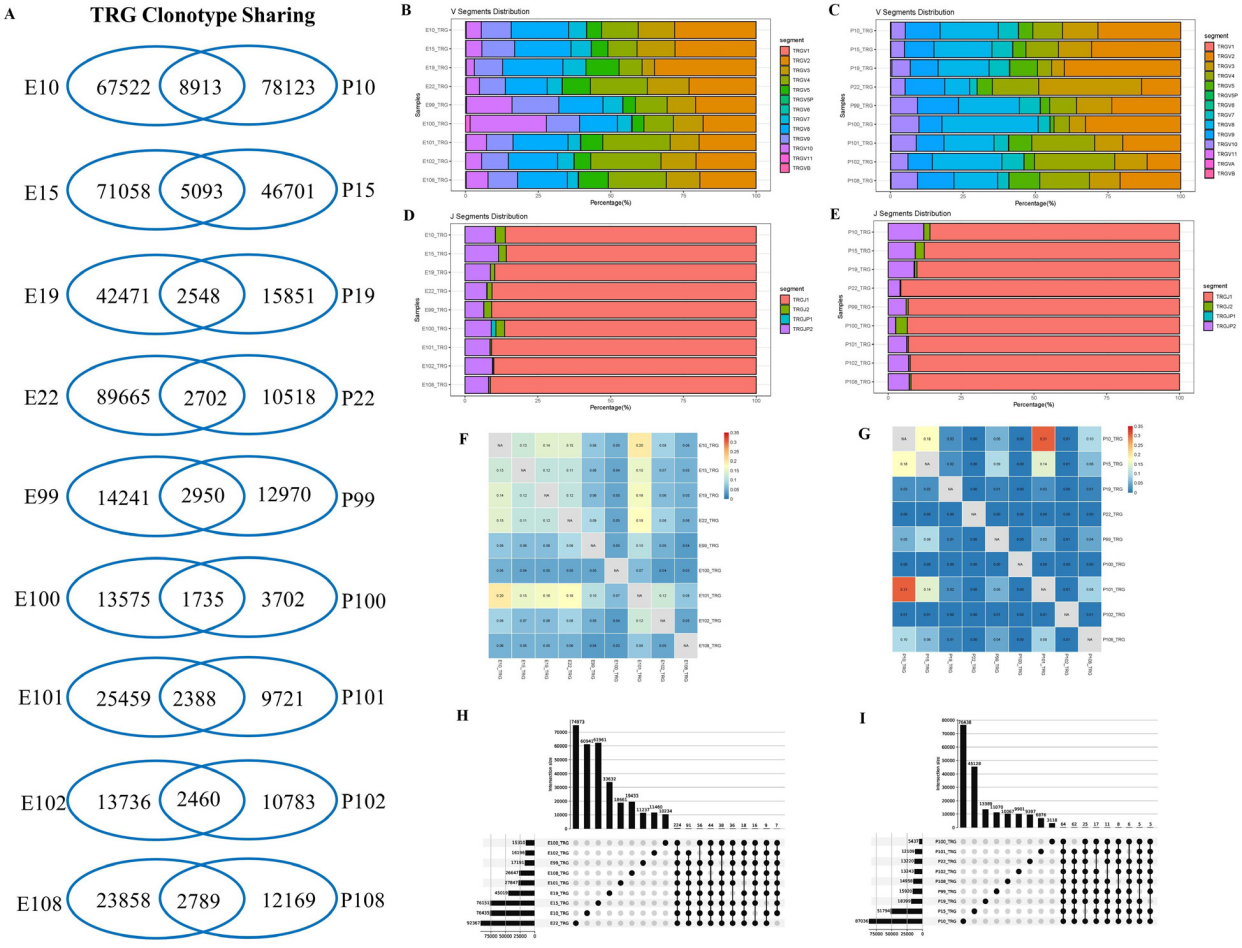
Characterization of T-cell receptor γ repertoires between the pleural effusion and blood in PLTB patients. **(A)** TCR sharing across the pleural effusion and blood in PLTB patients. Venn diagram shows the sharing of TRG clonotypes across the PE and blood. Values indicate the number of distinct or shared clonotypes. **(B, C)** The distribution of TRG V gene segments usage in the pleural effusion **(B)** and blood **(C)**. **(D, E)** The TRG J gene segments usage in the pleural effusion **(D)** and blood **(E)**. **(F, G)** Morisita index was used to assess the similarity of TRG repertoires between the pleural effusion **(F)** and blood **(G)** in PLTB patients. **(H, I)** Multi-Venn analysis of the same TRG clonotypes found in pleural effusion **(H)** or blood samples **(I)** in PLTB patients. The numbers shown on the left of the yellow bars mean the total clonotypes in each patient. The dots and connection lines mean the samples analyzed individually or across the selected samples, and the numbers on top of the black bars mean the unique clonotypes in each sample and the public clonotypes across the selected compared samples.

For TRG repertoires in PLTB patients, the data were similarly analyzed as the TRB repertoires (**Figure 3**; **Supplementary PPT Data File1**-**3**). The results indicate that all nine PLTB patients shared some TRG clones across the pleural effusion and blood (**Figure 3A**; **Supplementary Figure S3**). The patients have similar patterns of V or J segment distribution between the pleural effusion or blood (**Figures 3B, C**). However, we can still find that TRGV3 and TRGV8 have higher gene usage in the blood compared with the pleural effusions in patients 22 and 100 separately. The TRG analysis indicates that some samples have a higher TRG Morisita index, like E10 and E101, and P10 and P101, which are highly different from the TRB Morisita index among the samples. The multi-Venn analysis indicates that there are 224 TRG clones shared in all nine pleural effusion samples, and 64 TRG clones shared all across the nine blood samples (**Figures 3H, I**; **Supplementary Excel**).

### B cell repertoires and antibodies responses in PLTB patients

**Figure 4:**
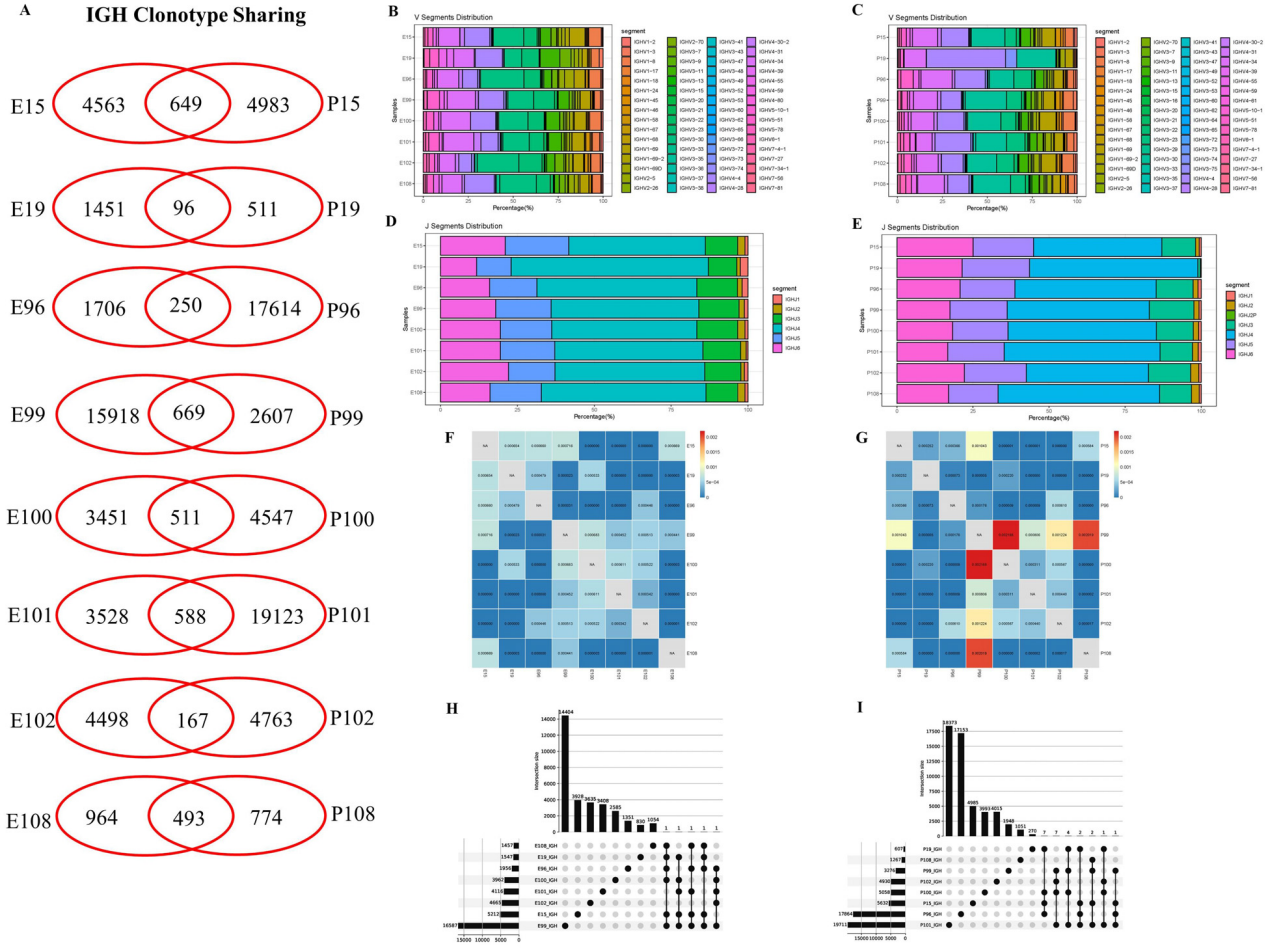
IGH clonotype sharing, V and J segment distribution, and IGH repertoire similarities between samples measured by Morisita index. **(A)** IGH clonotype sharing. Venn diagram shows the sharing of IGH clonotypes between the blood and pleural effusions of PLTB patients. Values indicate the numbers of distinct clonotypes unique to blood or pleural effusions, or sharing clonotypes between samples. **(B, C)** Antibody IGH V segment distributions in the blood and pleural effusion samples of the eight PLTB patients. **(D, E)** Antibody IGH J segment distributions in the blood and pleural effusion samples of the eight PLTB patients. **(F, G)** IGH repertoire similarities between samples measured by Morisita index. The similarities of IGH repertoires between pleural effusions and the blood of the eight PLTB patients. All metrics range from 0 to 1, in which 1 represents an identical TCR repertoire and 0 represents completely distinct TCR repertoires. **(H, I)** Multi-Venn analysis of the same IGH clonotypes found in pleural effusion **(H)** or blood samples **(I)** in PLTB patients. The numbers shown on the left of the yellow bars mean the total clonotypes in each patient. The dots and connection lines mean the samples analyzed individually or across the selected samples, and the numbers on top of the black bars mean the unique clonotypes in each sample and the public clonotypes across the selected compared samples.

**Figure 5:**
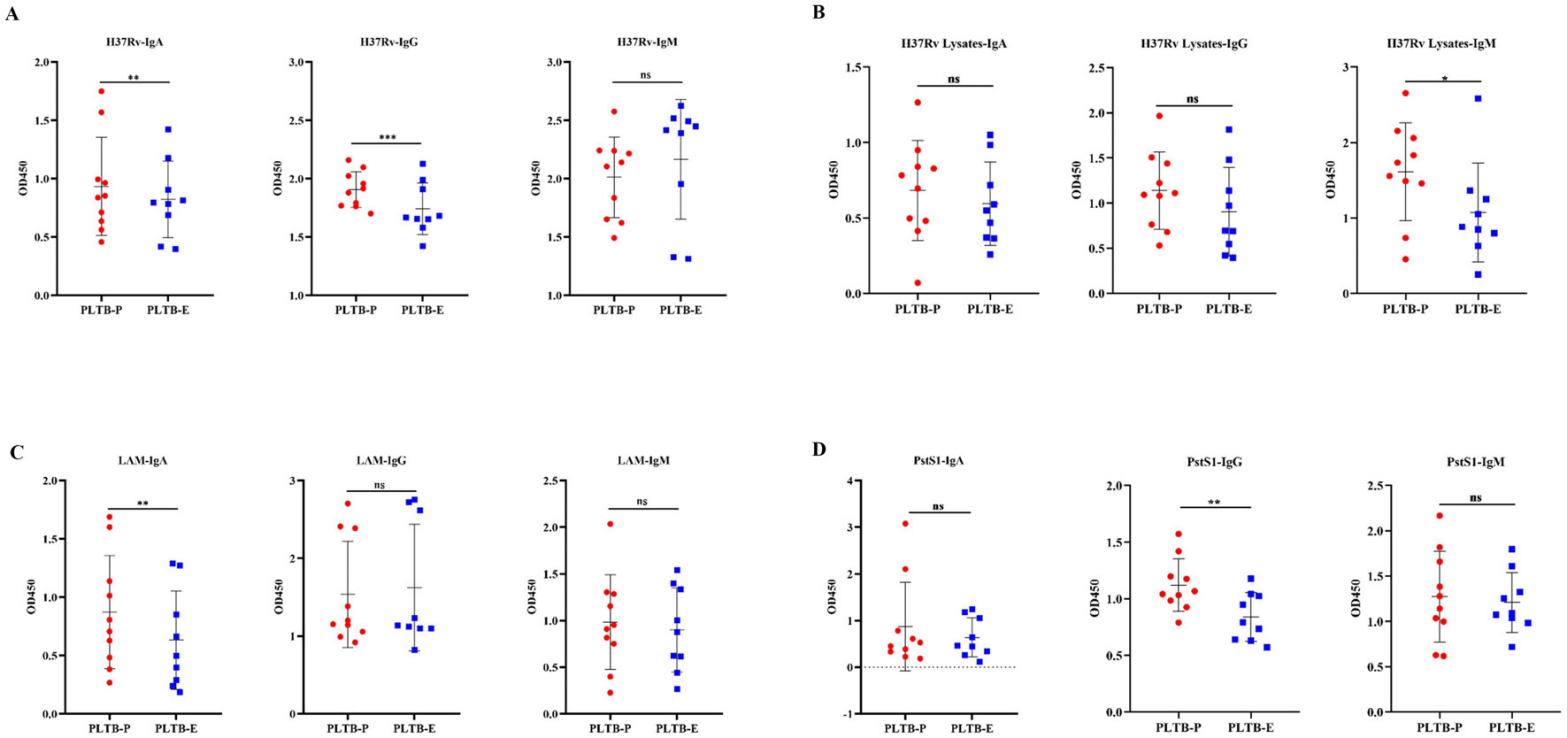
Binding activity of antibodies in plasma and pleural effusion of PLTB patients against different Mtb antigens. **(A–D)** Determination of the binding activity of IgA, IgG, and IgM antibodies in plasma (filled red circle) and pleural effusion (filled blue square) to the heat-killed H37Rv bacteria, H37Rv lysates, purified LAM, and PstS1 by ELISA, separately. OD450 was measured, and statistical analyses were performed by two-tailed T-tests (ns, no significance; *p < 0.05; **p < 0.01; ***p < 0.001).

In this study, BCR IGH repertoires in PLTB patients were also analyzed (**Figure 4**; **Supplementary PPT Data File1**-**3**). According to the results, we can find that all eight enrolled PLTB patients shared some IGH clones across the pleural effusions and blood samples, which indicates that the shared clones may be from the circulation of this patient (**Figure 4A**; **Supplementary Figure S3**). Most of the patients have similar V or J gene segment distribution across the pleural effusions and blood. Despite this, we can notice that in patient 19, the IGHV4 genes usage in blood was significantly higher compared with the pleural effusions, which indicates there are also different immune responses in individual PLTB patients (**Figures 4B–E**). The Morisita index indicates that there are shared IGH clones between some pleural effusions and blood samples, which may suggest that these clones are potential Mtb-specific IGH clones (**Figures 4F, G**). Two shared IGH clones are observed in seven pleural effusions and five are in five blood samples by multi-Venn analysis, respectively (**Figures 4H, I**; **Supplementary Excel**), which indicates that these antibodies may have roles in the patients and need to be further investigated. We also tested the IgA, IgG, and IgM antibody subtype responses against Mtb antigens by ELISA. The results indicate that there are different antibody responses to Mtb antigens in the blood and pleural effusions in PLTB patients (**Figures 5A–D**). The level of IgA antibody responses to whole H37Rv bacteria and LAM antigens in plasma is higher compared with pleural effusions of PLTB patients (**Figures 5A, C**), and the IgG responses to whole H37Rv and PstS1 in plasma are higher compared with in pleural effusions (**Figures 5A, D**). For IgM responses, the plasma has stronger antibody responses to H37Rv lysates than pleural effusion (**Figure 5B**).

### Identification of CDR3β by GLIPH2 and Mtb-specific TRB analysis

**Figure 6:**
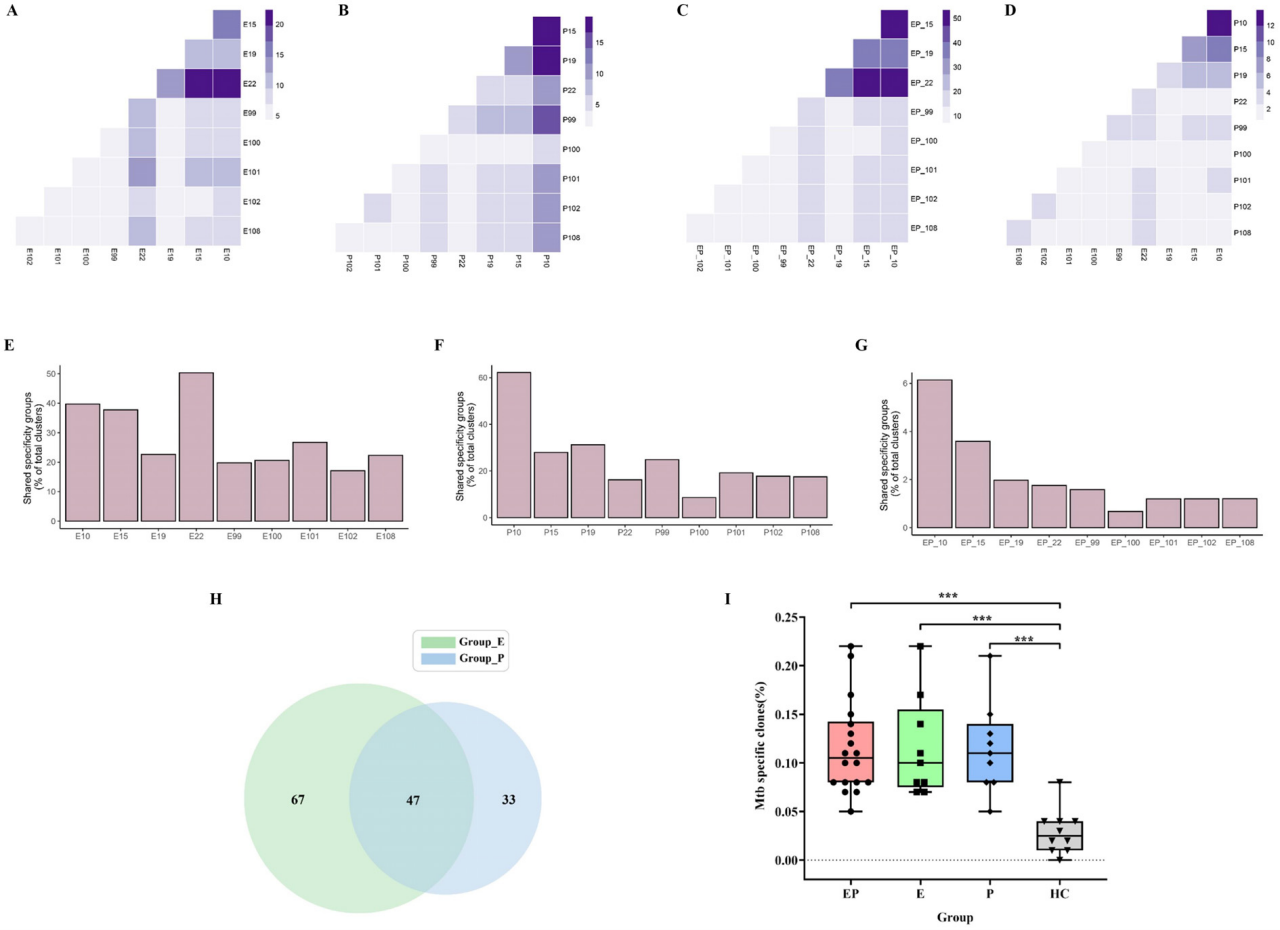
GLIPH2 analysis of TRB repertoires reveals Mtb-specific TCRs in PLTB patients. **(A–D)** Heatmap shows the shared TRB specificity groups in the pleural effusions **(A)** or blood **(B)**, or between the pleural effusions and blood **(C)**, or combination of pleural effusions and blood **(D)** in the specific pairs of PLTB patients as determined by GLIPH2 analysis. **(E–G)** Bar blot displays the proportions of the shared TRB specificity groups determined by GLIPH2 analysis in the pleural effusions **(E)**, blood **(F)**, and both **(G)**. The shared Mtb-specific TRB clones in the pleural effusion and blood of all PLTB patients by Venn analysis **(H)**. The comparison of Mtb-specific clones’ ratio between pleural effusion, blood, or both and HC **(I)**. Statistical analyses were performed using the ANOVA Kruskal–Wallis test (***p <0.001).

To identify disease-relevant T-cell receptors (TCRs) with shared antigen specificity, we analyzed 276,056 TCRβ chain sequences from the pleural effusion and the blood of nine patients with pleural tuberculosis using GLIPH2 (**Supplementary Figure S2**). According to the results, the pleural effusion and blood groups have 11,668 and 7,537 TCR clusters, respectively. A total of 15,913 high-quality shared specificity groups with a set of specific filtering criteria were identified in nine PLTB patients. We further identified 1,147 specificity groups with evidence of clonal expansion, and 296 of these were enriched in pleural effusion compared with blood samples (**Supplementary Figure S2**). Therefore, the CDR3β of these 296 specificity groups is inferred to recognize the Mtb-specific antigens, which need to be further determined. GLIPH2 is an algorithm developed for clustering TCRs with a high probability of recognizing the same epitope into specificity groups, which is based on conserved motifs and similarity levels of CDR3β. Additionally, the provision of HLA typing results can provide the prediction of HLA restrictions in specific TCR clusters (**Supplementary Table S1**). The results indicate that the pleural effusion of pairs of patients 10 and 22, 15 and 22, and 10 and 15 has the highest overlap proportions of shared specificity groups (22.4%, 20.1%, and 17.1%), and the blood of pairs of patients 10 and 15, 10 and 19, and 10 and 99 have the highest overlap (19.2%, 17.6%, and 14.3%) (**Figures 6A, B**). Moreover, patients 10 and 15, 10 and 22, and 15 and 22 have the highest overlap of specific TCR clusters (53.5%, 53.3%, and 48.1%) when combining the blood and pleural effusion specificity groups (**Figure 6C**). In addition, we also analyze the shared specific clusters between pleural effusion and blood of the patients, and the results indicate that the same patient usually has a higher overlap between pleural effusion and blood compared with different patients (**Figure 6D**). Patient 22 has the highest specificity clusters among all the clusters in pleural effusions of all PLTB patients, which accounts for 50.4% (**Figure 6E**). The analysis indicates that patient 10 has the most shared TCR specificity clusters, which account for 62.2% of the blood of all PLTB patients, and patient 100 has the lowest shared clusters at only 8.6%. Interestingly, patient 10 has the most shared specificity clusters among all the clusters in both the pleural effusion and blood of all the patients (6.1%) (**Figures 6F, G**). The results indicate that the shared specificity clusters have high proportions in the pleural effusions or blood, which are quite different from the specificity clusters in both the pleural effusions and blood. The results also suggest that the TCR specificity clusters in the pleural effusion and the blood are quite different, although there are some overlapping clusters between the pleural effusions and blood. The Mtb-specific TRB clones in each sample were identified, and 47 clones were shared by pleural effusion and blood of all the patients (**Figure 6H**; **Supplementary Excel Table S2**). Importantly, the ratio of Mtb-specific TRB clones in the blood, pleural effusion, or both of the PLTB patients is significantly higher compared with the health control (HC) group (**Supplementary Excel Table S3**), which suggests that the patients have the specific T-cell responses against Mtb, and the responses happened in both the blood and the pleural effusion (**Figure 6I**).

## Discussion

Tuberculosis is still the deadliest infectious disease around the world (1). It is urgent to develop novel and more effective vaccines for TB control. PLTB as one common extra-pulmonary TB is still a big challenge and has aroused more attention; however, the initiation, progression, and interaction of Mtb and the host are still unclear. In this study, we did a deep sequencing and performed a comprehensive analysis of the TCR and BCR repertoires in PLTB patients, which display a landscape of immune responses in PLTB.

T-cell immunity is important for Mtb control. The decrease of CD4+ T cells in HIV patients makes them susceptible to Mtb infections (50). Th1 CD4+ T cells can simultaneously secrete IFN-γ, TNF-α, and IL-2 which can affect the innate immune response especially the macrophage response for controlling intracellular Mtb. CD8+ T cells also have a protective role during *M. tuberculosis* infection, and they can be specific for Mtb antigens which can recognize infected macrophages, produce cytokines, have cytotoxicity to the infected cells, and directly kill Mtb (51). Furthermore, Th17 cells have the protection roles against Mtb infection by attracting and activating the neutrophils (52, 53). The other T-cell subset (unconventional T cells) such as CD1-restricted T cells and γδ T cells also have protective roles in Mtb infection. Studies indicate that immunization of CD1 transgenic mice with Mtb lipids or alive Mtb can arouse the lipid-specific immune response (54). Since patients with tuberculosis pleurisy have a relatively effective immune response against Mtb (3, 18, 19), more and more studies have researched and characterized T-cell immunity in PLTB patients. One study indicates that CFP-10-specific CD4(+) and CD8(+) T cells in tubercular pleural fluid have biased usage of TCR Vβ9, Vβ12, or Vβ7.2 (55). Another study used the high-throughput deep sequencing methods to analyze the TCRB repertoires of an untreated pleural tuberculosis patient, and the results indicated that the TRBV20-1 family and TRBV20-1/TRBJ1-5 gene combination had a dominant expression in PEMCs, but not in PBMCs of the patient (56). Recent studies used single-cell RNA sequencing (scRNA-seq) to characterize the T-cell immunity in tuberculosis pleural effusion (57, 58). The lineage tracking in one study indicated that the CD4 + T- and CD8+ T-cell populations with distinct effector functions expanding at pleural sites and granzyme K-expressing CD8 T cells were preferentially enriched and clonally expanded in pleural effusion (57). Another study used scRNA-seq to analyze and find the differences in immune cell responses in tuberculosis pleural effusion and non-TPE (58). The studies have provided important evidence that T cells are involved in the pathogenesis of PLTB and can improve our understanding of local TB immunopathogenesis. In our study, we used high-throughput DNA sequencing to analyze and characterize the TCRβ and TCRγ repertoires in pleural effusion and blood. The results indicate that there are similar patterns of TRB V or J segment distribution in the pleural effusion and blood, and no differences in TRB/TRG clonality and Shannon index between the pleural effusion or blood in PLTB patients were observed (**Supplementary Figure S3**), but we can still find differences in individuals. The frequencies of the TRBV5-3 and TRBJ2-3 segment usage in the pleural effusion are significantly higher compared with the blood in patient 19. TRGV3 and TRGV8 usage in the blood is more abundant in comparison with the pleural effusions in patients 22 and 100 separately. These results may suggest that the more abundant segments or clones might be of importance in the differential diagnosis of PLTB in the clinical setting and have potential biological effects on the PLTB progression. The activation of γδ T cells can be induced rapidly following mycobacterial infections, and γδ T cells can protect against tuberculosis by secreting cytokines and promoting anti-TB immune responses including both innate and adaptive immunity (59–62). In our study, the Morisita index indicates the similarities of TRG clones in the pleural effusion and blood of each patient is significantly higher compared with TRB clones. The results may suggest that γδ T cells should play important roles during Mtb infection in PLTB patients. In this study, we also identified the sharing of TRB and TRG clones in pleural effusion and blood, which may suggest that these shared clones may be attracted by Mtb at the early stage of infection in pleural effusion and originated from the blood, and whether these clones target Mtb or have protective roles needs to be further characterized and investigated. Moreover, Mtb-specific TRB clones were determined by VJ mapping to VDJdb and McPAS-TCR databases, and these Mtb-specific clones of PLTB patients have shown significant differences compared with the Health people, which suggests that these clones may have functional roles or have potential to be biomarkers for TB diagnosis.

B cells have important roles against Mtb infections, and increasing evidence indicates that antibodies have protective roles against TB (11–17). B cells expressing high-affinity BCRs can be selected by T follicular helper (Tfh) cells, which provide the required signal for B-cell differentiation (63). Antigen-specific B cells can enhance cytokine production and localize TFH-like cells within granuloma-associated lymphoid tissue (GrALT) by interacting with programmed cell death 1 (PD-1) and its ligand PD-L1, which can mediate Mtb control in both mice and macaques (64). Therefore, B cells as well as T cells and their synergistic effects are very important in Mtb infections, and it is worth investigating and characterizing the B cells’ immune responses in TB patients. In this study, we did the IGH deep sequencing of B cells in the pleural effusion and blood and identified the antibodies titer to the Mtb antigens. The Morisita index has shown the overlapping IGH clones among the patients, which suggests that the antibodies’ immune responses aroused by Mtb infections are similar in some PLTB patients and antibodies may have important roles in PLTB progression. The shared clones can be found in both the pleural effusion and blood of PLTB patients. The shared IGH clones identified by Venn analysis indicate that these clones may have potential roles in Mtb infections and need to be further investigated. The IGH clonality and Shannon index between the pleural effusion or blood in PLTB patients have no significance (**Supplementary Figure S3**). However, the different B cells’ immune responses can be found in individual PLTB patients. For example, the IGHV4 gene usage in the blood of patient 19 is significantly higher compared with the pleural effusion. These differences may be related to the different immunity and clinical status of PLTB patients (65). Moreover, the antibody titer results indicate that antibody responses targeting different Mtb antigens can be found in both the pleural effusion and blood, which indicates that antibodies may play important roles in Mtb initiation and progression in PLTB patients. This may be due to the migration of antigen-specific B lymphocytes at the site of infection from the peripheral blood of PLTB patients. These results are also consistent with the BCR repertoire analysis that there are shared IGH clones between the blood and pleural effusion. The differences in antibody titers to the antigens in pleural effusion and blood may suggest that the B cells have heterogeneous responses in pleural effusion and the blood, and the roles and their differences need to be further investigated.

High-throughput sequencing(HTS) is an innovative and good technology that can be an important tool for researchers precisely studying the adaptive immune response. For HTS repertoire analysis, both genomic DNA and RNA can be used for the library establishment and sample sequencing (66–68). One reason that we chose to use gDNA as a starting template for immune repertoire sequencing is that it is easier to obtain stable DNA from either fresh or frozen samples. Another reason is that DNA repertoire sequencing can reflect the quantity of the repertoire better, since each cell may only have one copy of the successfully rearranged V(D)J and help detect the low-frequency clones and determine the relative abundance of clonotypes (66–70). Furthermore, bulk sequencing costs less, is faster, and is less laborious; therefore, it is usually used for investigating and characterizing the immune repertoires in patients and healthy people (66–70). In this study, bulk DNA sequencing was carried out to analyze and characterize the immune repertoires of PLTB patients, and this can give important suggestions for further investigating the subpopulation cells or even a single immune cell by scRNA-seq (66–70). For example, it is worth determining the paired heavy chain and light chains of Mtb-specific TCRs or BCRs by scRNA-seq, and identifying their protection against Mtb infections in future studies.

Above all, in this study, we used high-throughput sequencing technology to characterize the adaptive immune responses in PLTB patients, investigate the similarities and differences, and analyze the shared clones in pleural effusion and blood, which give us more precise insights into the TB immune responses and help us for TB diagnosis development and finding novel immunogens for vaccine development.

## Data Availability

The data presented in the study are deposited in the OMIX repository, accession number OMIX007914.

## Supplementary material

Supplementary Figure S1 The purity analysis of T cells isolated from the blood and pleural effusion of PLTB patients by flow cytometry. The PBMCs and PEMCs were separately isolated from the whole blood and pleural effusion, and then the T cells were purified from the PBMCs and PEMCs by negative selection. The purified T cells were stained by FITC staining anti-CD3 antibody and the samples were analyzed by BD C6 FACS machine. **(A, C)** The T cells isolated from the blood **(A)** and pleural effusion **(C)** were not stained by CD3-FITC antibody as the control; **(B, D)** The T cells isolated from the blood **(B)** or pleural effusion **(D)** were stained by CD3-FITC antibody.

Supplementary Figure S2 Specificity inference pipeline.

Supplementary Figure S3 The comparison of clonality and Shannon index between the pleural effusion or blood. TRB **(A, B)**; TRG **(C, D)**; IGH **(E, F)**.

Supplementary Table S1 HLA alleles of the PLTB patients enrolled in this study.

SUPPLEMENTARY Excel Table S1 The list of shared clones of TRB, TRG, and IGH between the blood and pleural effusion of the PLTB patients.

SUPPLEMENTARY Excel Table S2 The list of shared Mtb-specific clones between the blood and pleural effusion of the PLTB patients.

SUPPLEMENTARY Excel Table S3 The features and TRB statistics of 10 healthy controls.

Supplementary PPT data file 1 TRB, TRG, and IGH CDR3 length frequency in the pleural effusion and blood of each PLTB patient.

Supplementary PPT data file 2 TRB, TRG, and IGH clonality frequencies of the pleural effusion and blood in each PLTB patient are displayed by donut charts.

Supplementary PPT data file 3 TRB, TRG, and IGH VJ combination usage in the pleural effusion and blood of each PLTB patient.
