# Supplementary figures and images for: T-cell receptor and B-cell receptor repertoires profiling in pleural tuberculosis
Source: Front Immunol. 2024 Nov 27;15:1473486. doi: 10.3389/fimmu.2024.1473486 (PMC11632106; doi:10.3389/fimmu.2024.1473486)

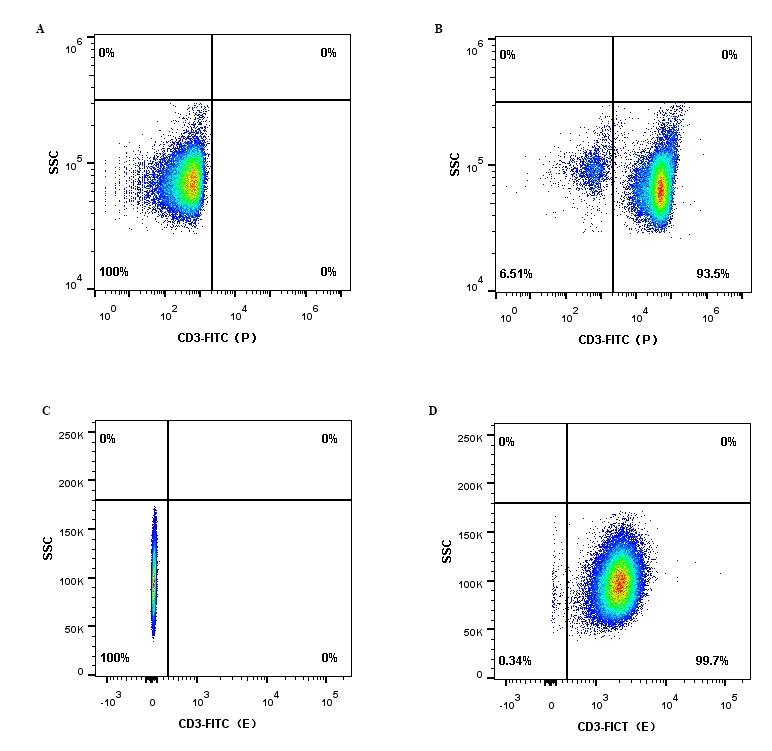

Supplement: Supplementary Figure S1 — The purity analysis of T cells isolated from the blood and pleural effusion of PLTB patients by flow cytometry. The PBMCs and PEMCs were separately isolated from the whole blood and pleural effusion, and then the T cells were purified from the PBMCs and PEMCs by negative selection. The purified T cells were stained by FITC staining anti-CD3 antibody and the samples were analyzed by BD C6 FACS machine. (A, C) The T cells isolated from the blood (A) and pleural effusion (C) were not stained by CD3-FITC antibody as the control; (B, D) The T cells isolated from the blood (B) or pleural effusion (D) were stained by CD3-FITC antibody. [file Image1.tif]

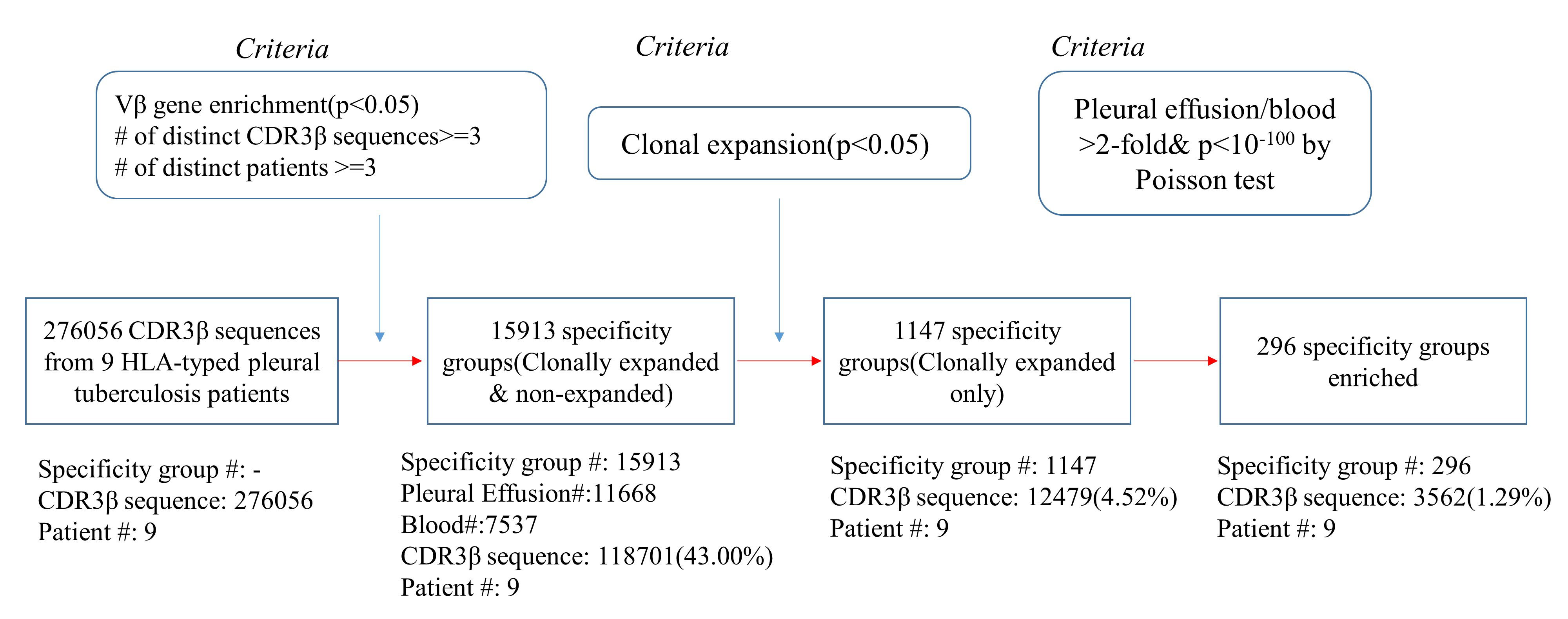

Supplement: Supplementary Figure S2 — Specificity inference pipeline. [file Image2.tif]

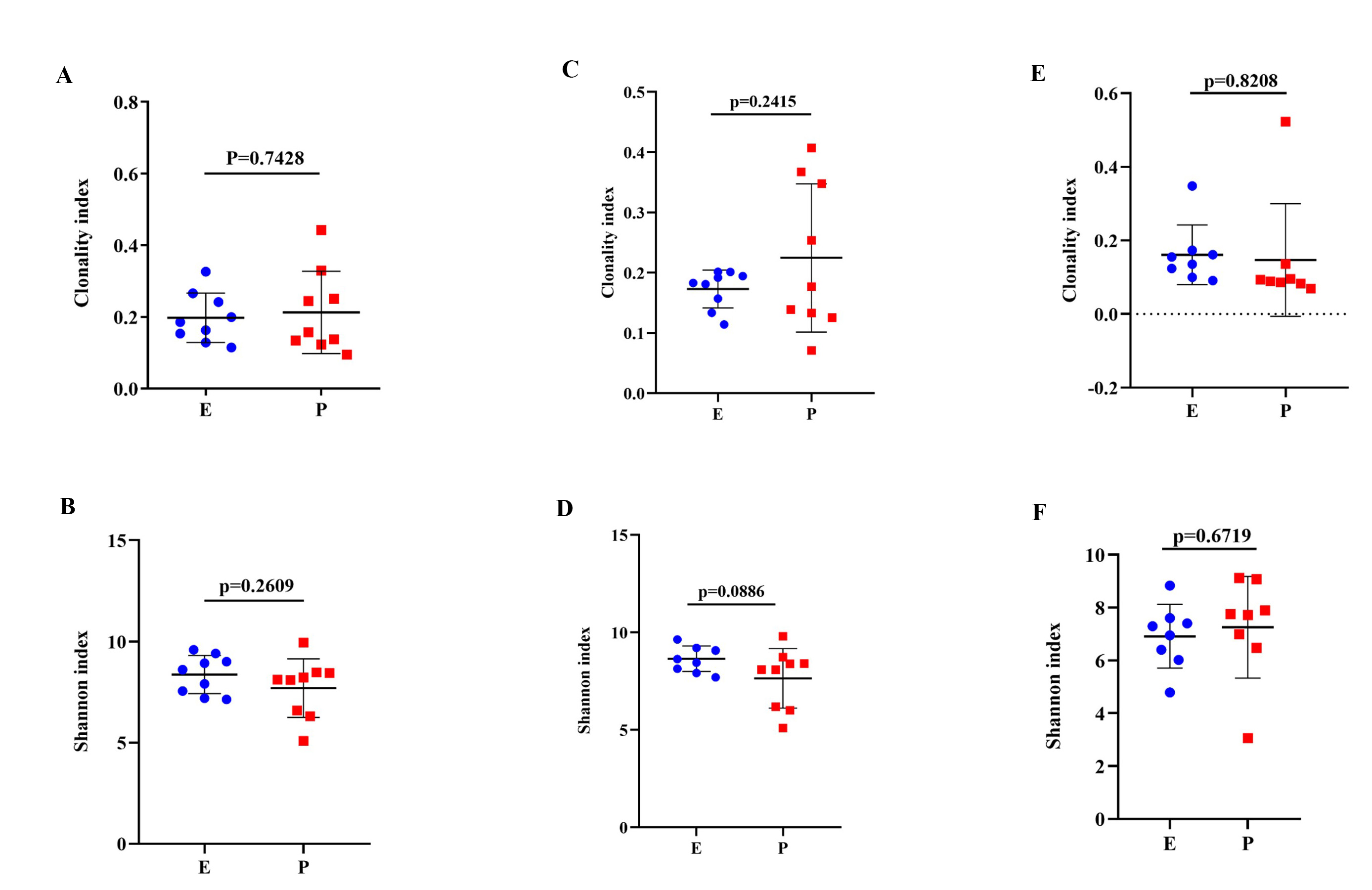

Supplement: Supplementary Figure S3 — The comparison of clonality and Shannon index between the pleural effusion or blood. TRB (A, B); TRG (C, D); IGH (E, F). [file Image3.tif]
