# Supplementary material for: T-cell receptor and B-cell receptor repertoires profiling in pleural tuberculosis
Source: Front Immunol. 2024 Nov 27;15:1473486. doi: 10.3389/fimmu.2024.1473486 (PMC11632106; doi:10.3389/fimmu.2024.1473486)
Supplement: Supplementary Table S1 — HLA alleles of the PLTB patients enrolled in this study. [file Table1.docx]

| **Patient** | **HLA-A^*^** | **HLA-B^*^** | **HLA-C^*^** | **HLA-DRB1^*^** | **HLA-DQB1^*^** | **HLA-DPB1*** |
| --- | --- | --- | --- | --- | --- | --- |
| 10 | A*11:01; A*24:10 | B*18:02; B*40:01 | C*08:03; C*08:22 | DRB1*15:01; DRB1*15:01 | DQB1*03:01; DQB1*05:01 | DPB1*13:01; DPB1*135:01 |
| 15 | A*11:01; A*24:02 | B*40:01; B*55:02 | C*07:02; C*15:02 | DRB1*12:02; DRB1*13:02 | DQB1*03:01; DQB1*06:04 | DPB1*05:01; DPB1*05:01 |
| 19 | A*01:01; A*11:01 | B*15:02; B*37:01 | C*06:02; C*08:01 | DRB1*04:05; DRB1*10:01 | DQB1*04:01; DQB1*05:01 | DPB1*02:01; DPB1*05:01 |
| 22 | A*02:01; A*24:02 | B*51:01; B*52:01 | C*12:02; C*14:02 | DRB1*14:54; DRB1*15:02 | DQB1*05:02; DQB1*06:01 | DPB1*02:01; DPB1*03:01 |
| 99 | A*02:07; A*11:02 | B*15:01; B*46:01 | C*01:02; C*04:01 | DRB1*04:05; DRB1*09:01 | DQB1*03:02; DQB1*03:03 | DPB1*02:01; DPB1*13:01 |
| 100 | A*02:01; A*03:01 | B*15:11; B*40:01 | C*03:03; C*07:02 | DRB1*12:01; DRB1*15:02 | DQB1*06:01; DQB1*03:01 | DPB1*02:02; DPB1*04:01 |
| 101 | A*01:01; A*02:01 | B*35:03; B*51:02 | C*01:02; C*04:01 | DRB1*13:01; DRB1*14:54 | DQB1*05:02; DQB1*06:03 | DPB1*05:01; DPB1*05:01 |
| 102 | A*24:02; A*33:03 | B*40:06; B*44:03 | C*08:01; C*14:03 | DRB1*09:01; DRB1*13:02 | DQB1*06:04; DQB1*03:01 | DPB1*02:01; DPB1*04:01 |
| 108 | A*11:01; A*24:02 | B*07:02; B*15:11 | C*03:03; C*07:02 | DRB1*15:01; DRB1*15:01 | DQB1*06:02; DQB1*06:02 | DPB1*04:01; DPB1*04:02 |

**Supplemental Table S1. HLA alleles of the PLTB patients enrolled in this study**
