# Supplementary material for: T-cell receptor and B-cell receptor repertoires profiling in pleural tuberculosis
Source: Front Immunol. 2024 Nov 27;15:1473486. doi: 10.3389/fimmu.2024.1473486 (PMC11632106; doi:10.3389/fimmu.2024.1473486)
Supplement: Supplementary PPT data file 1 — TRB, TRG, and IGH CDR3 length frequency in the pleural effusion and blood of each PLTB patient. [file Presentation1.ppt]

## Slide 1
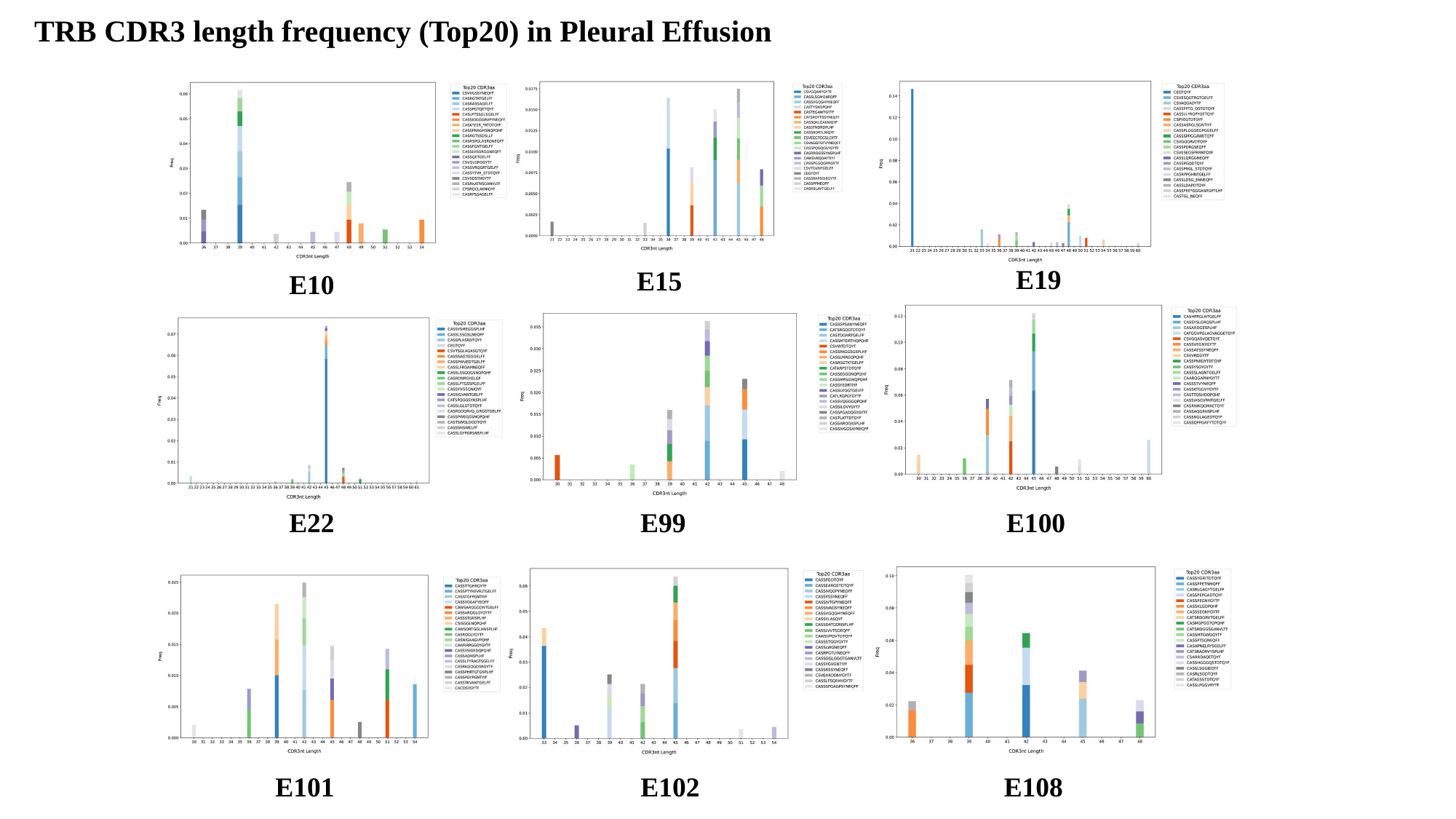

TRB CDR3 length frequency (Top20) in Pleural Effusion
E19
E15
E10
E22
E99
E100
E101
E102
E108

## Slide 2
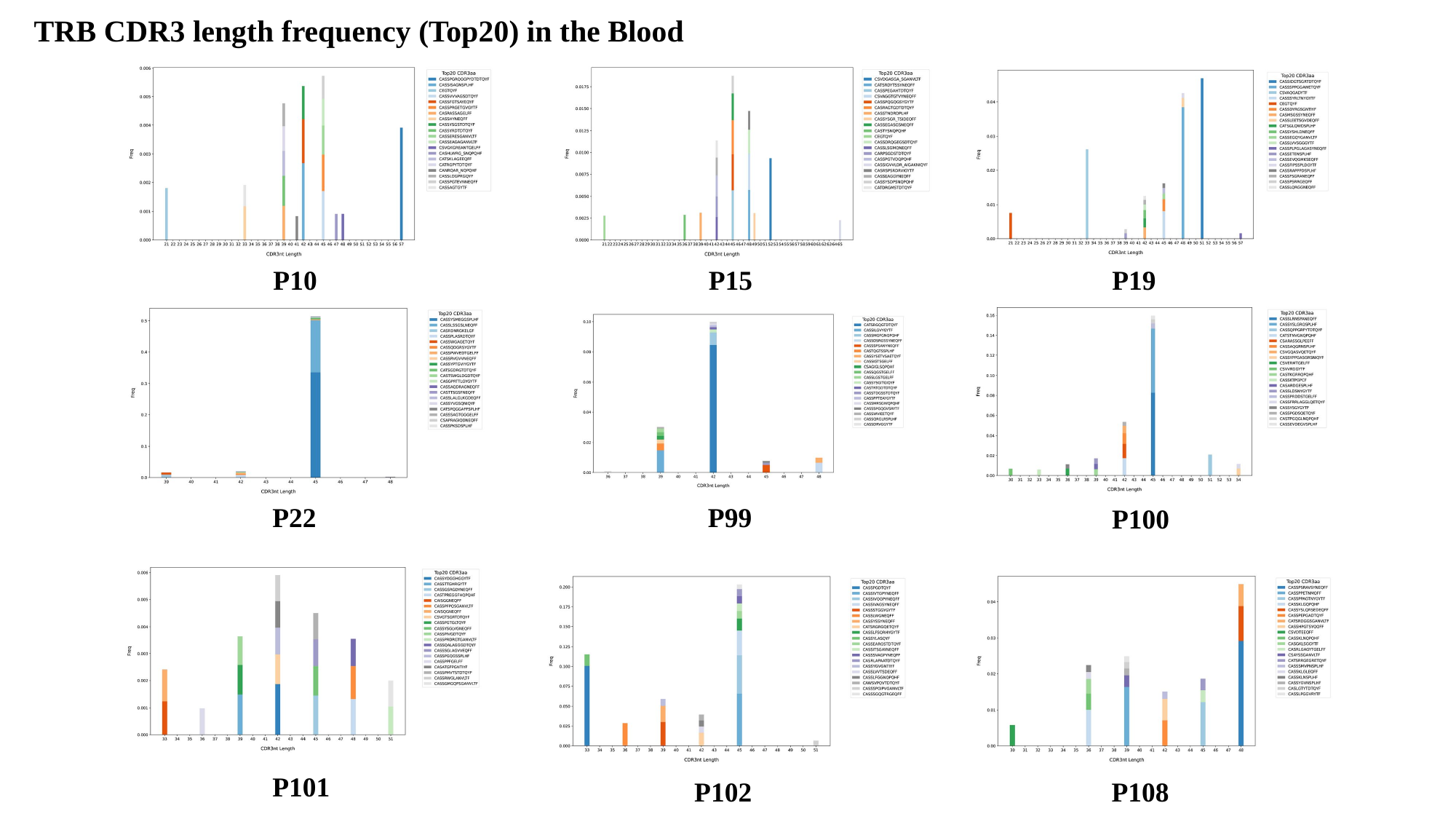

TRB CDR3 length frequency (Top20) in the Blood
P10
P15
P19
P22
P99
P100
P101
P102
P108

## Slide 3
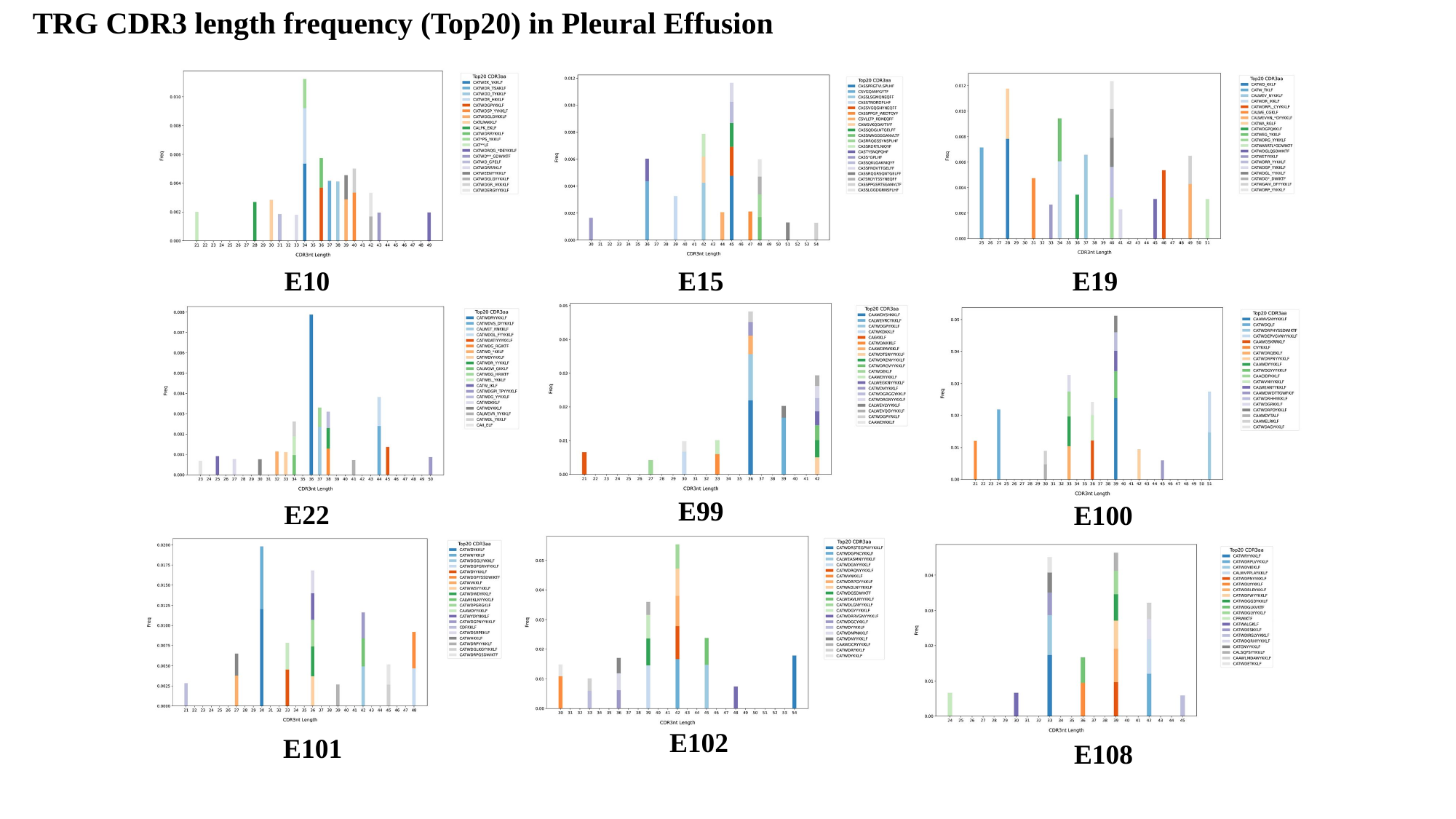

TRG CDR3 length frequency (Top20) in Pleural Effusion
E10
E15
E19
E99
E22
E100
E102
E101
E108

## Slide 4
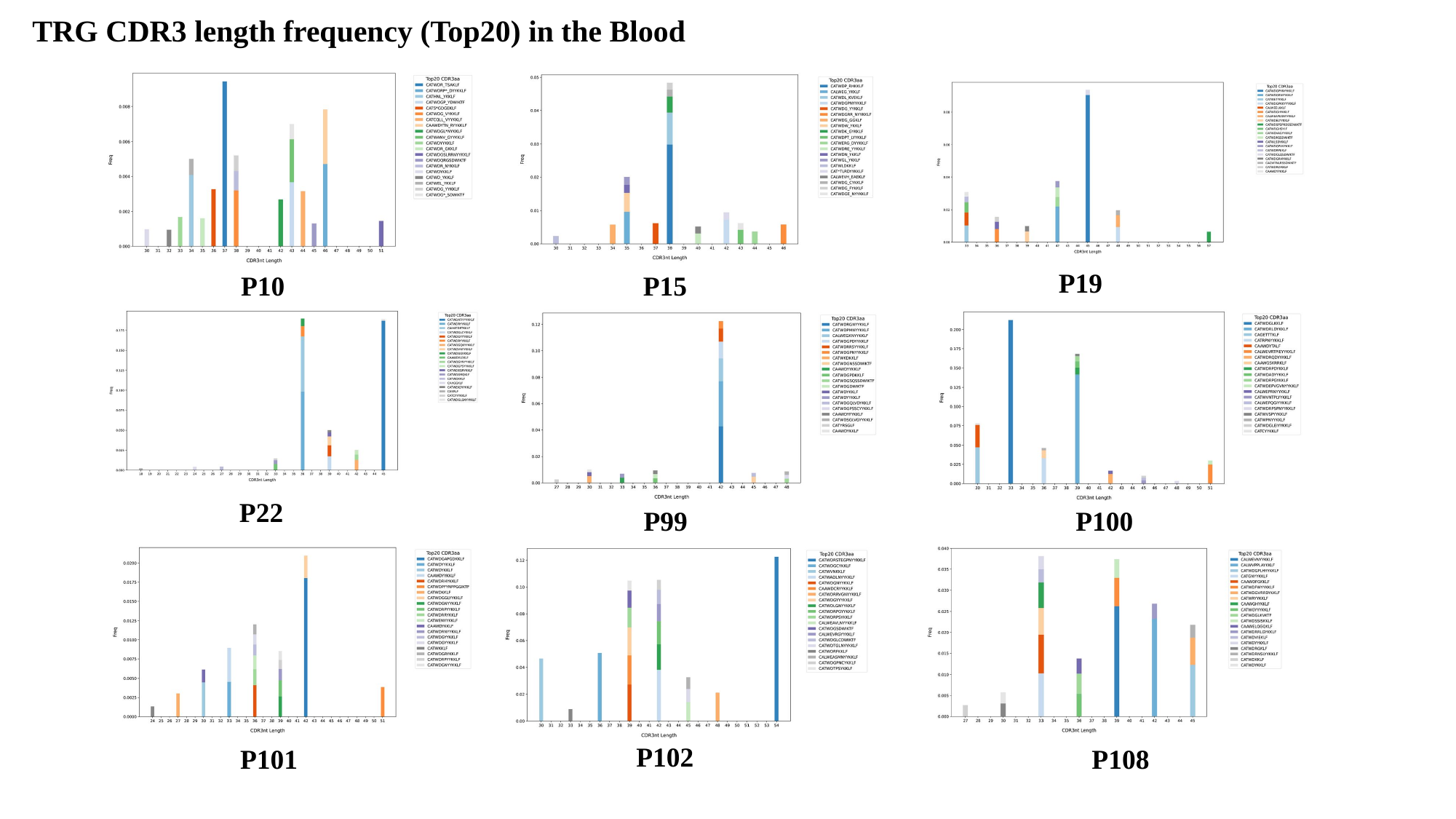

TRG CDR3 length frequency (Top20) in the Blood
P19
P10
P15
P22
P99
P100
P102
P101
P108

## Slide 5
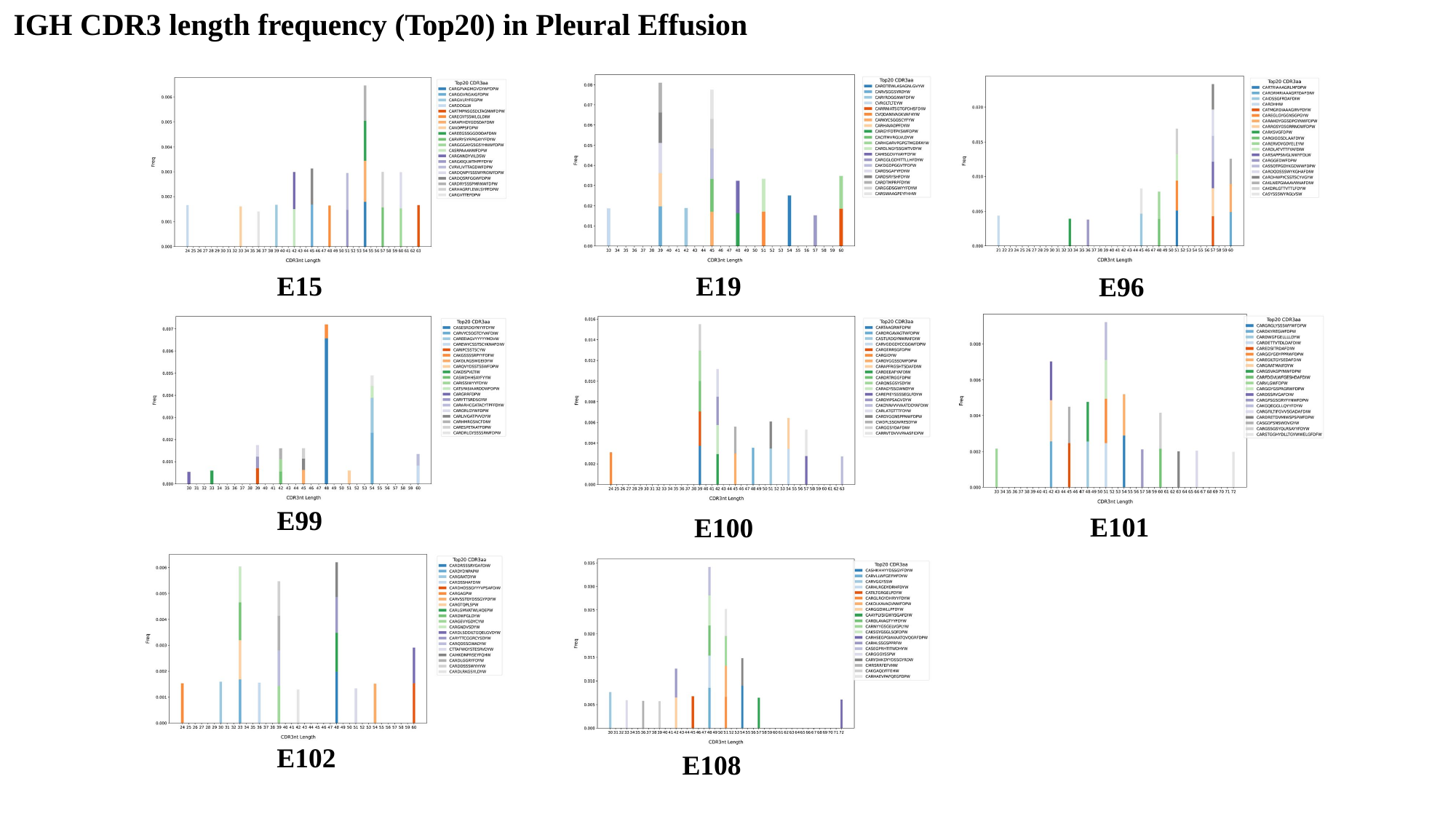

IGH CDR3 length frequency (Top20) in Pleural Effusion
E15
E19
E96
E99
E101
E100
E102
E108

## Slide 6
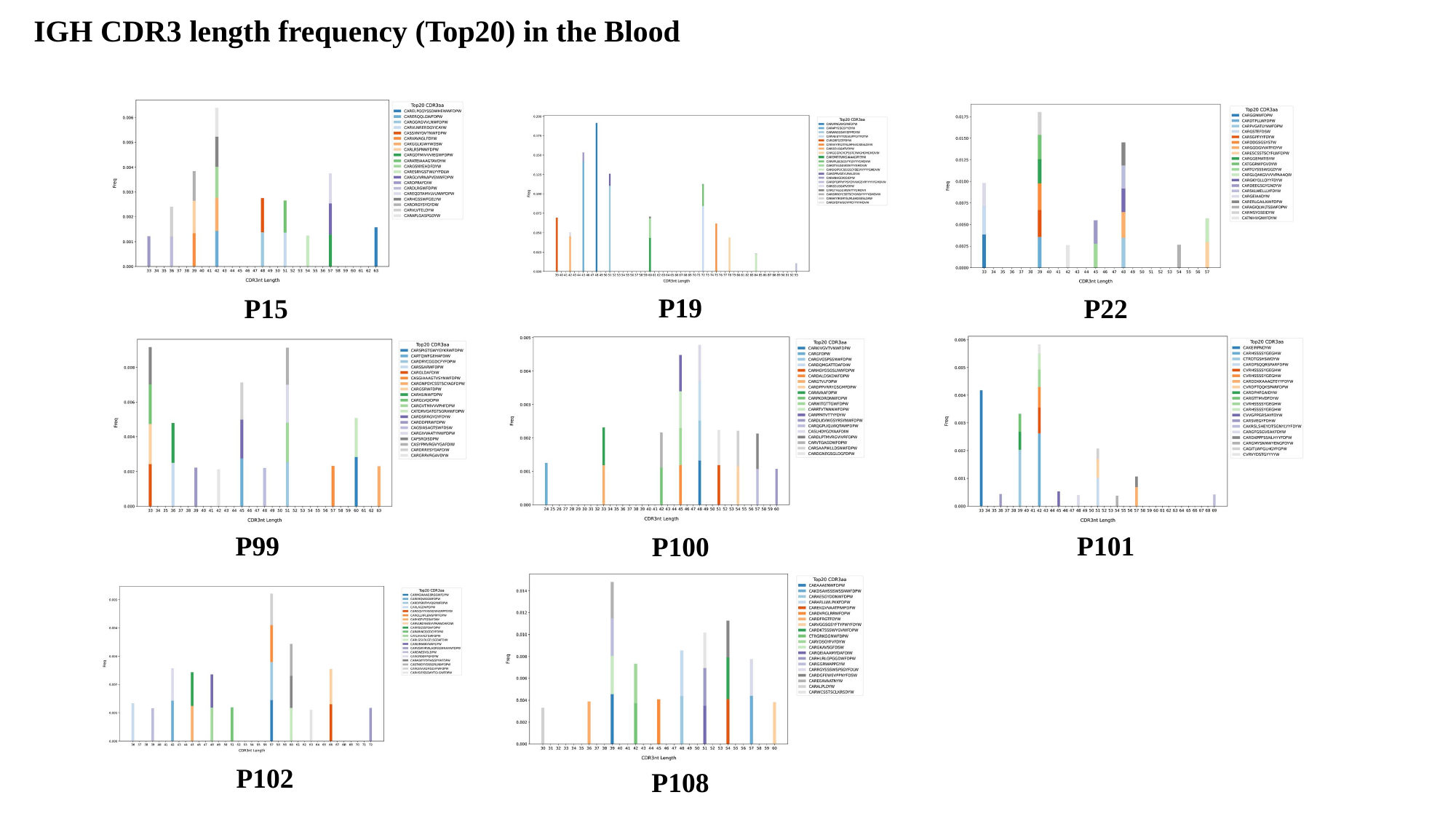

IGH CDR3 length frequency (Top20) in the Blood
P19
P15
P22
P99
P101
P100
P102
P108
