# Supplementary material for: T-cell receptor and B-cell receptor repertoires profiling in pleural tuberculosis
Source: Front Immunol. 2024 Nov 27;15:1473486. doi: 10.3389/fimmu.2024.1473486 (PMC11632106; doi:10.3389/fimmu.2024.1473486)
Supplement: Supplementary PPT data file 2 — TRB, TRG, and IGH clonality frequencies of the pleural effusion and blood in each PLTB patient are displayed by donut charts. [file Presentation2.ppt]

## Slide 1
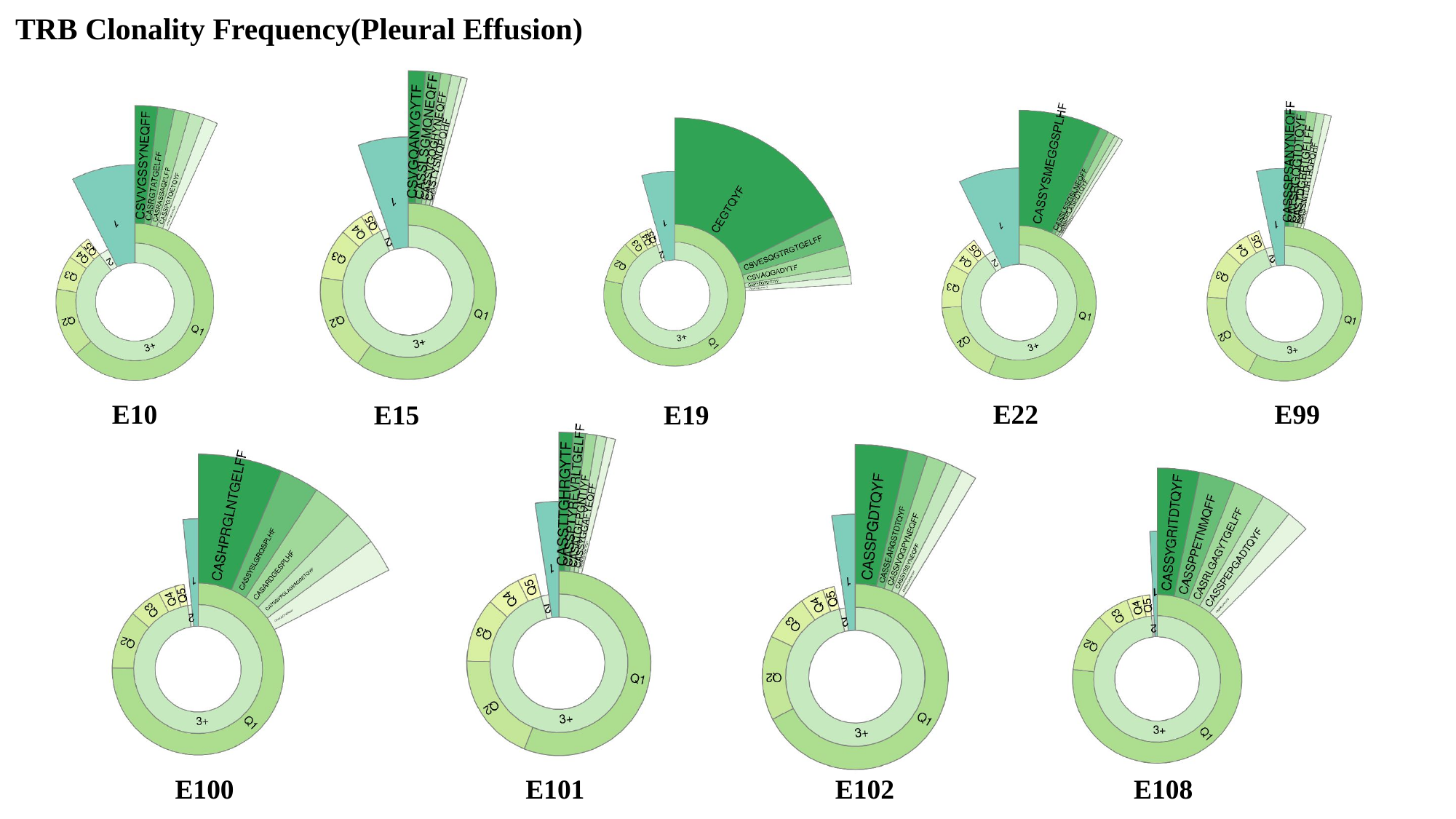

TRB Clonality Frequency(Pleural Effusion)
E10
E22
E99
E15
E19
E100
E101
E102
E108

## Slide 2
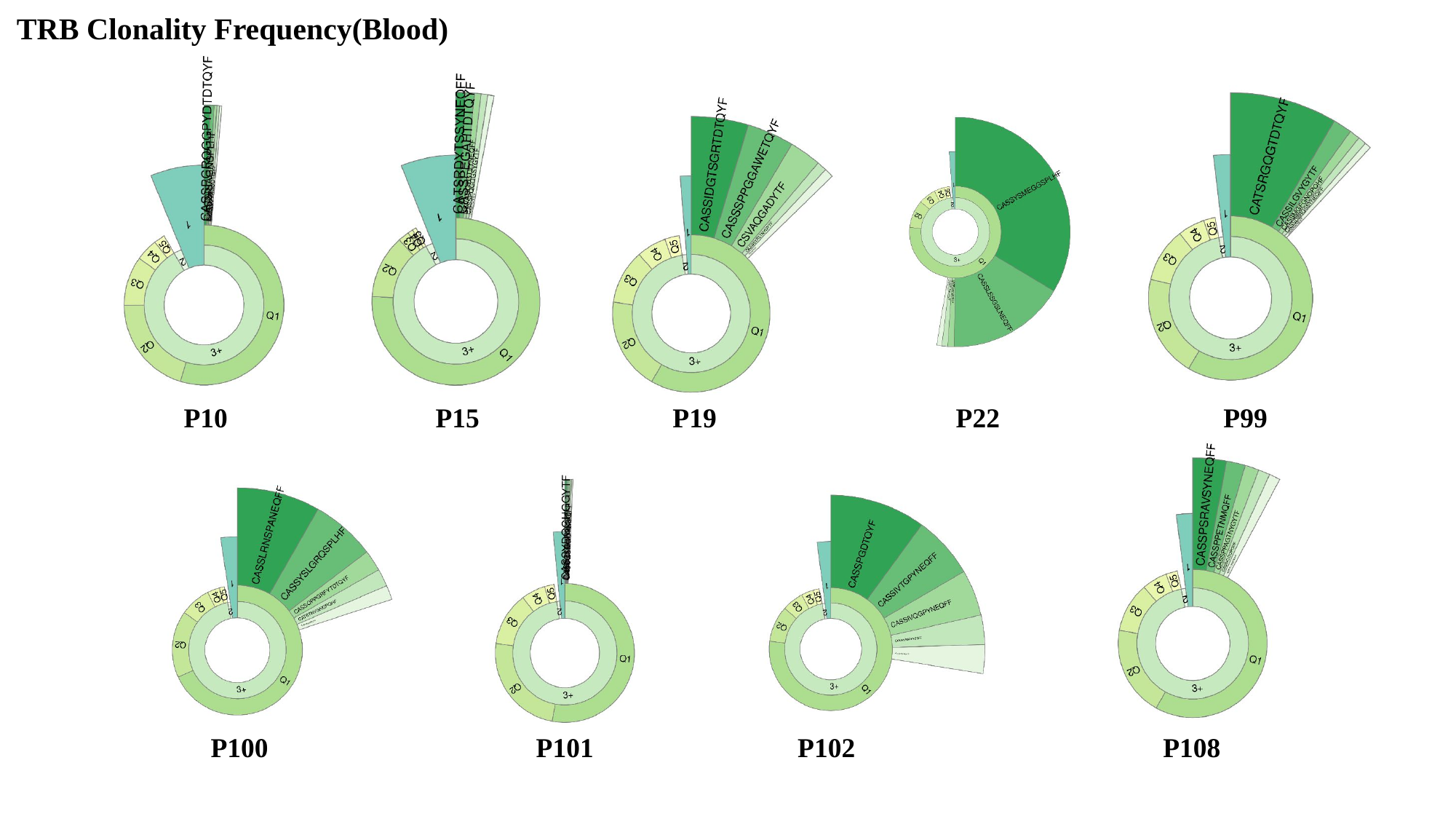

TRB Clonality Frequency(Blood)
P10
P19
P22
P99
P15
P100
P101
P102
P108

## Slide 3
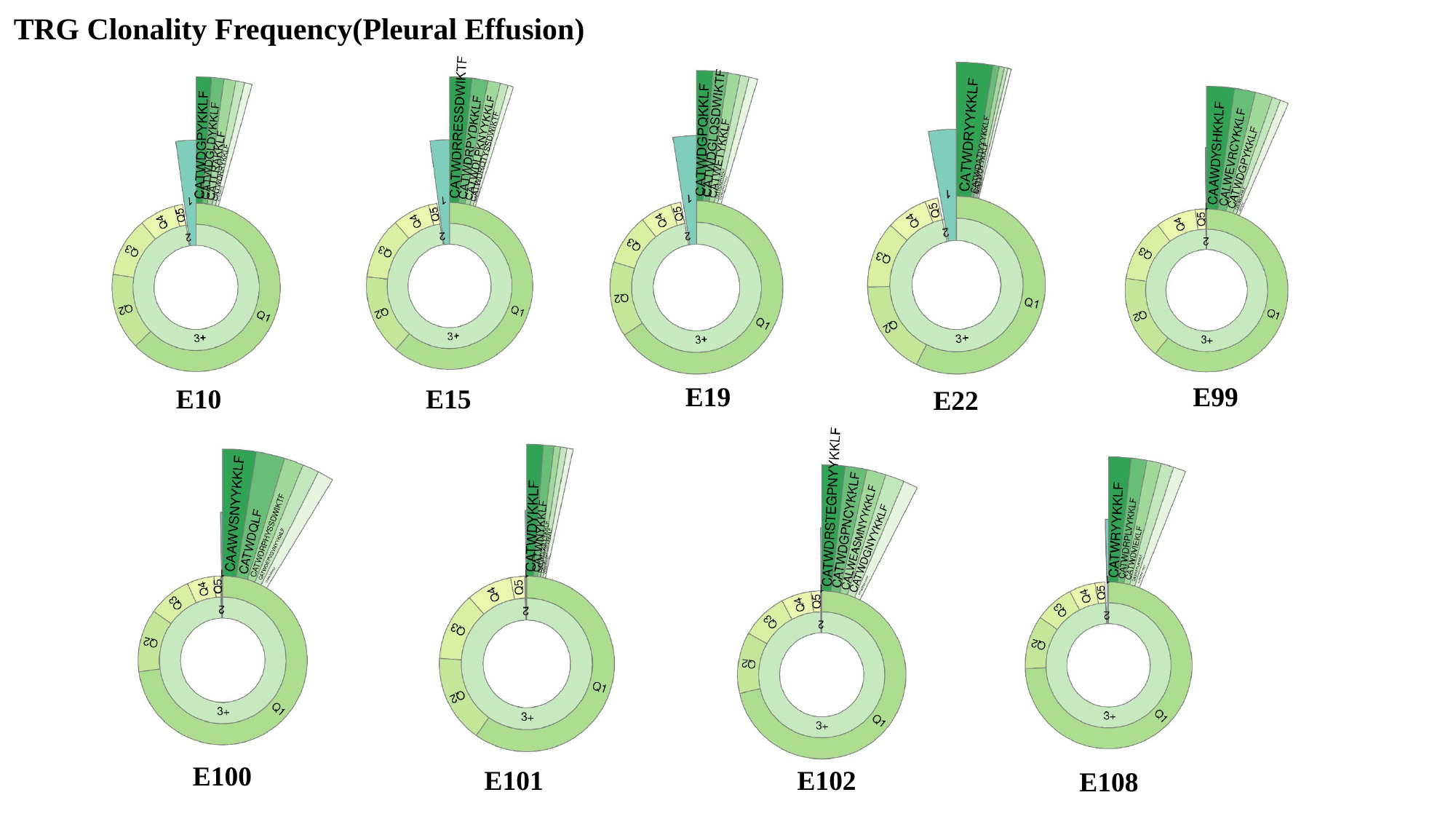

TRG Clonality Frequency(Pleural Effusion)
E19
E99
E10
E15
E22
E100
E101
E102
E108

## Slide 4
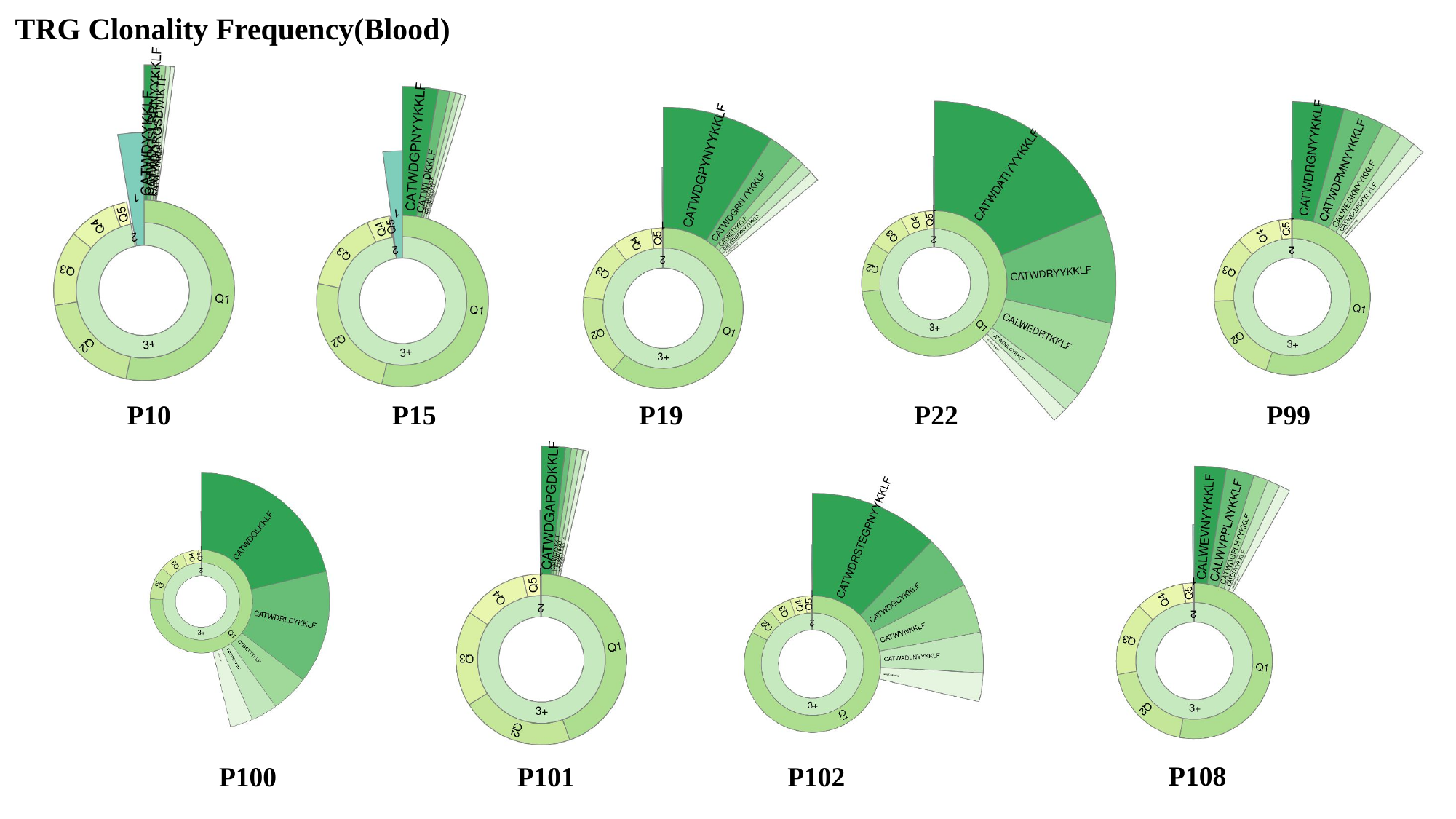

TRG Clonality Frequency(Blood)
P10
P15
P19
P22
P99
P108
P100
P101
P102

## Slide 5
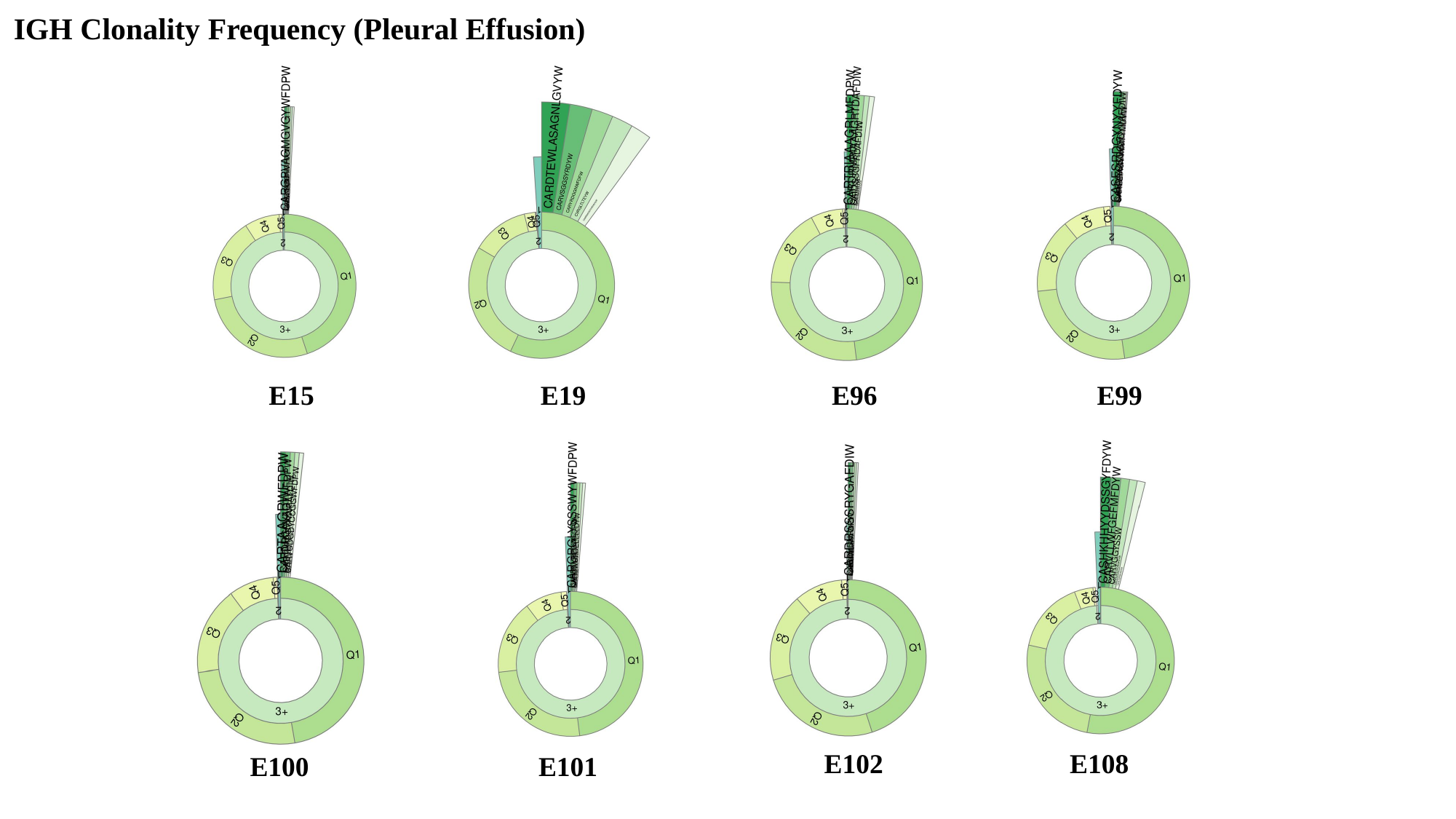

IGH Clonality Frequency (Pleural Effusion)
E15
E19
E96
E99
E102
E108
E100
E101

## Slide 6
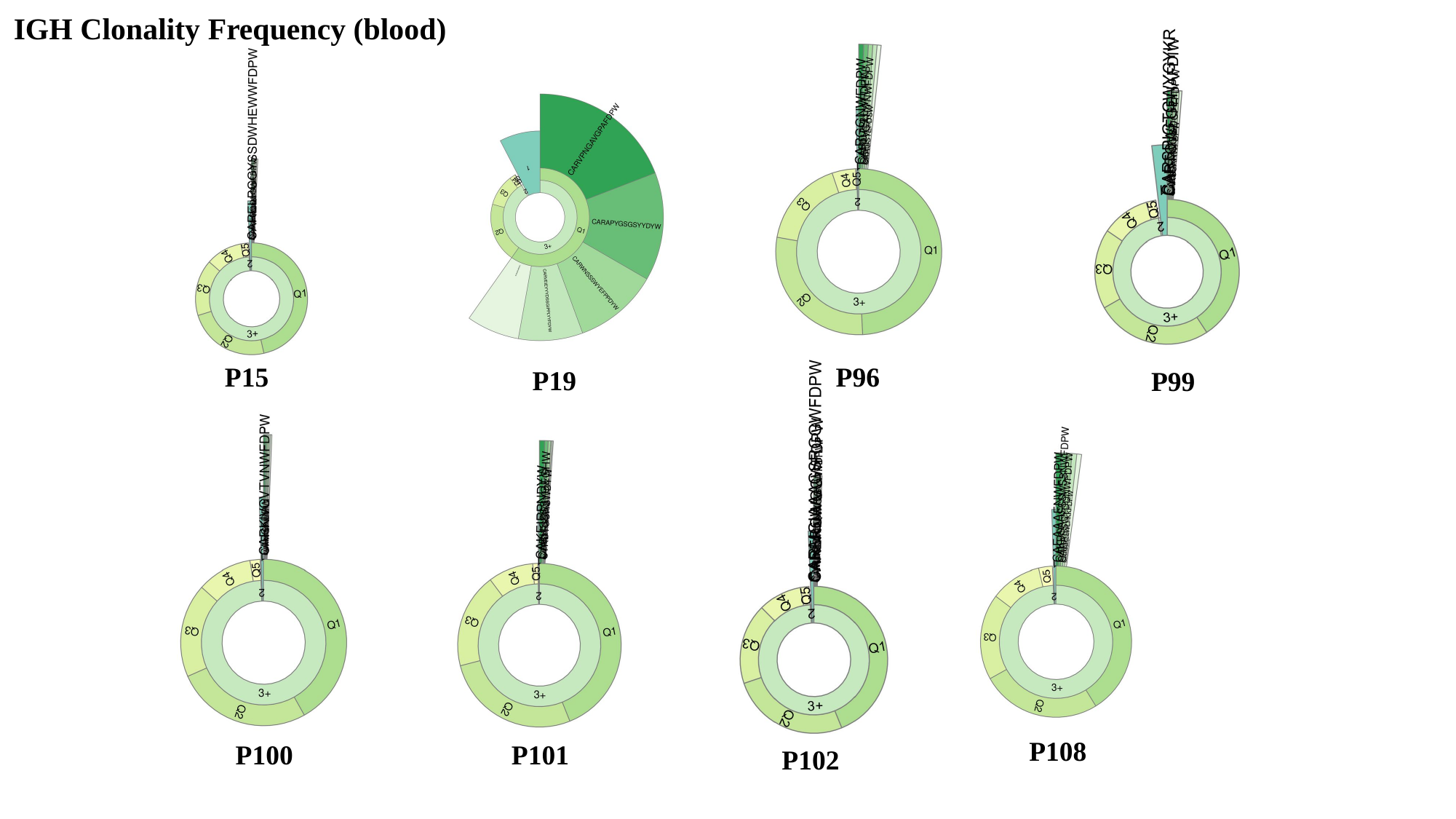

IGH Clonality Frequency (blood)
P15
P96
P19
P99
P108
P100
P101
P102
