# Supplementary material for: T-cell receptor and B-cell receptor repertoires profiling in pleural tuberculosis
Source: Front Immunol. 2024 Nov 27;15:1473486. doi: 10.3389/fimmu.2024.1473486 (PMC11632106; doi:10.3389/fimmu.2024.1473486)
Supplement: Supplementary PPT data file 3 — TRB, TRG, and IGH VJ combination usage in the pleural effusion and blood of each PLTB patient. [file Presentation3.ppt]

## Slide 1
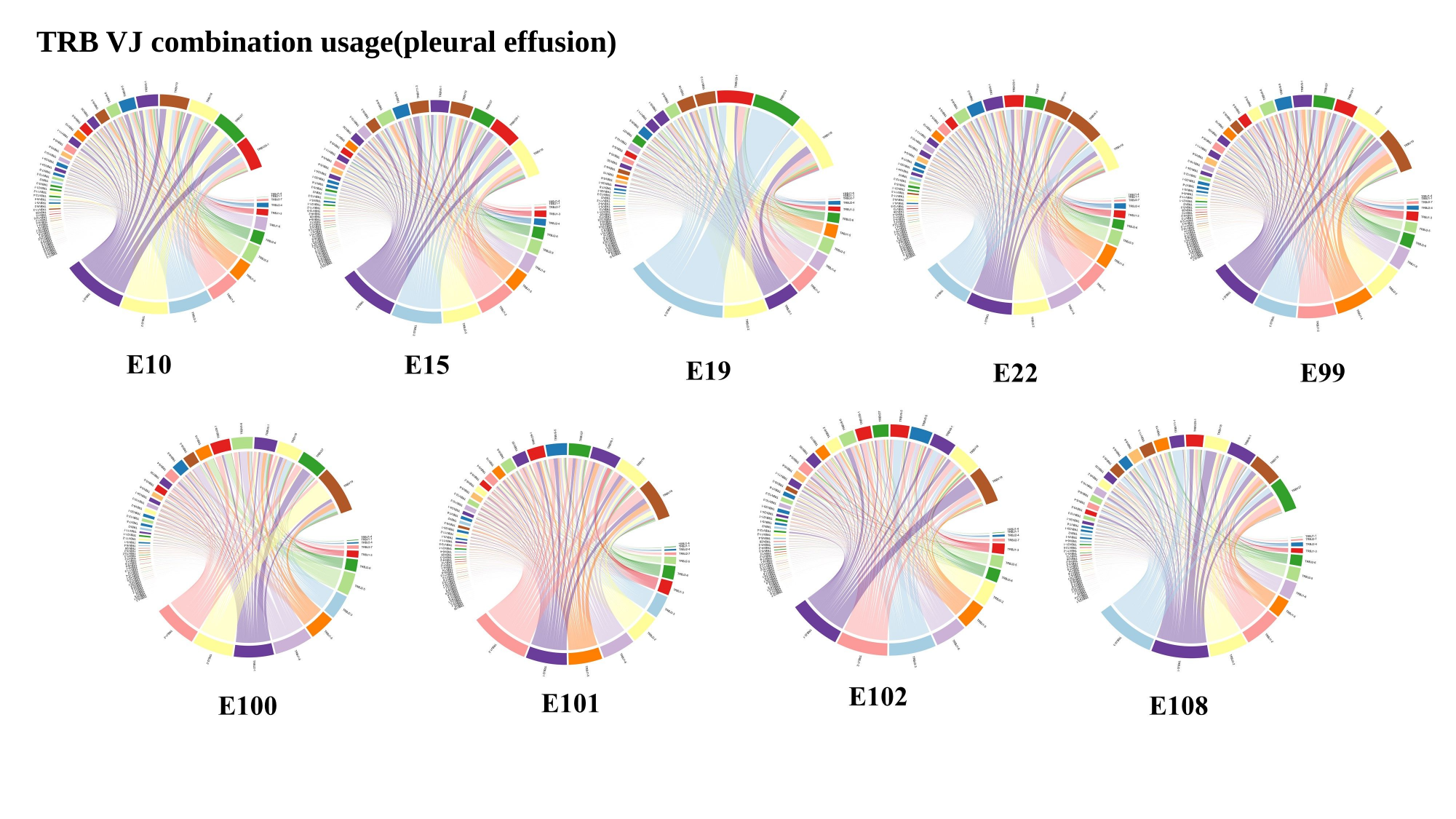

TRB VJ combination usage(pleural effusion)

## Slide 2
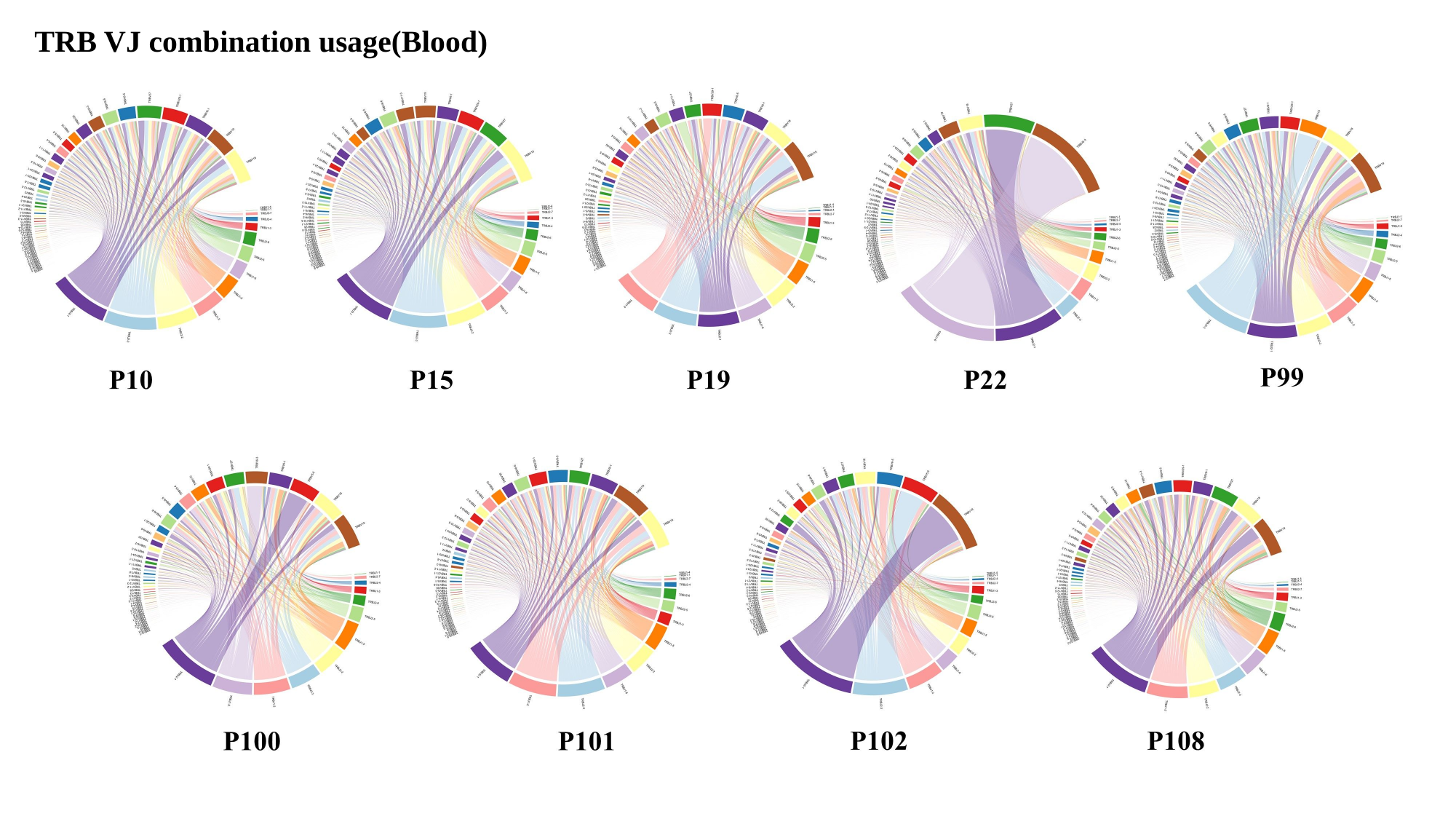

TRB VJ combination usage(Blood)

## Slide 3
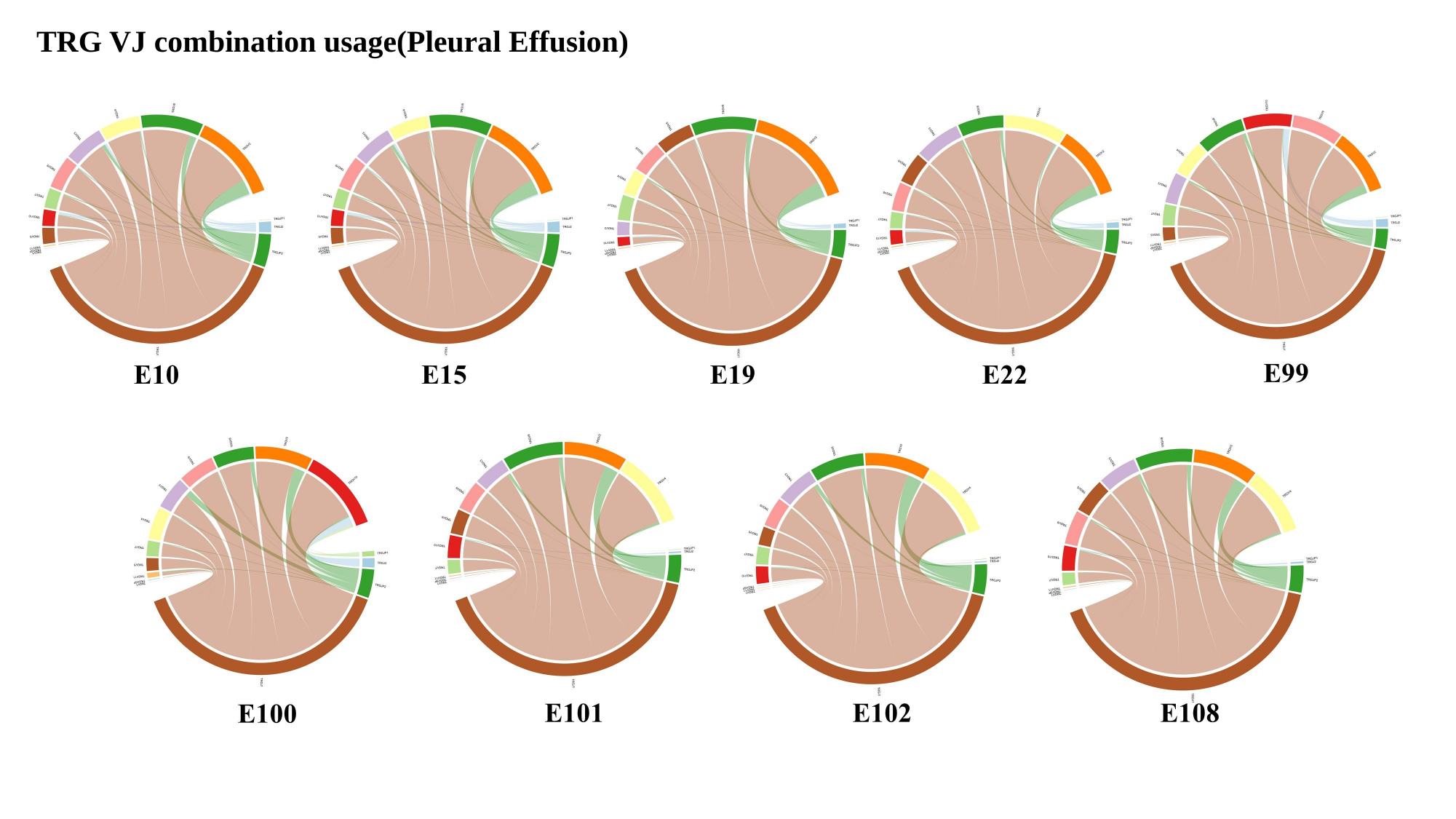

TRG VJ combination usage(Pleural Effusion)

## Slide 4
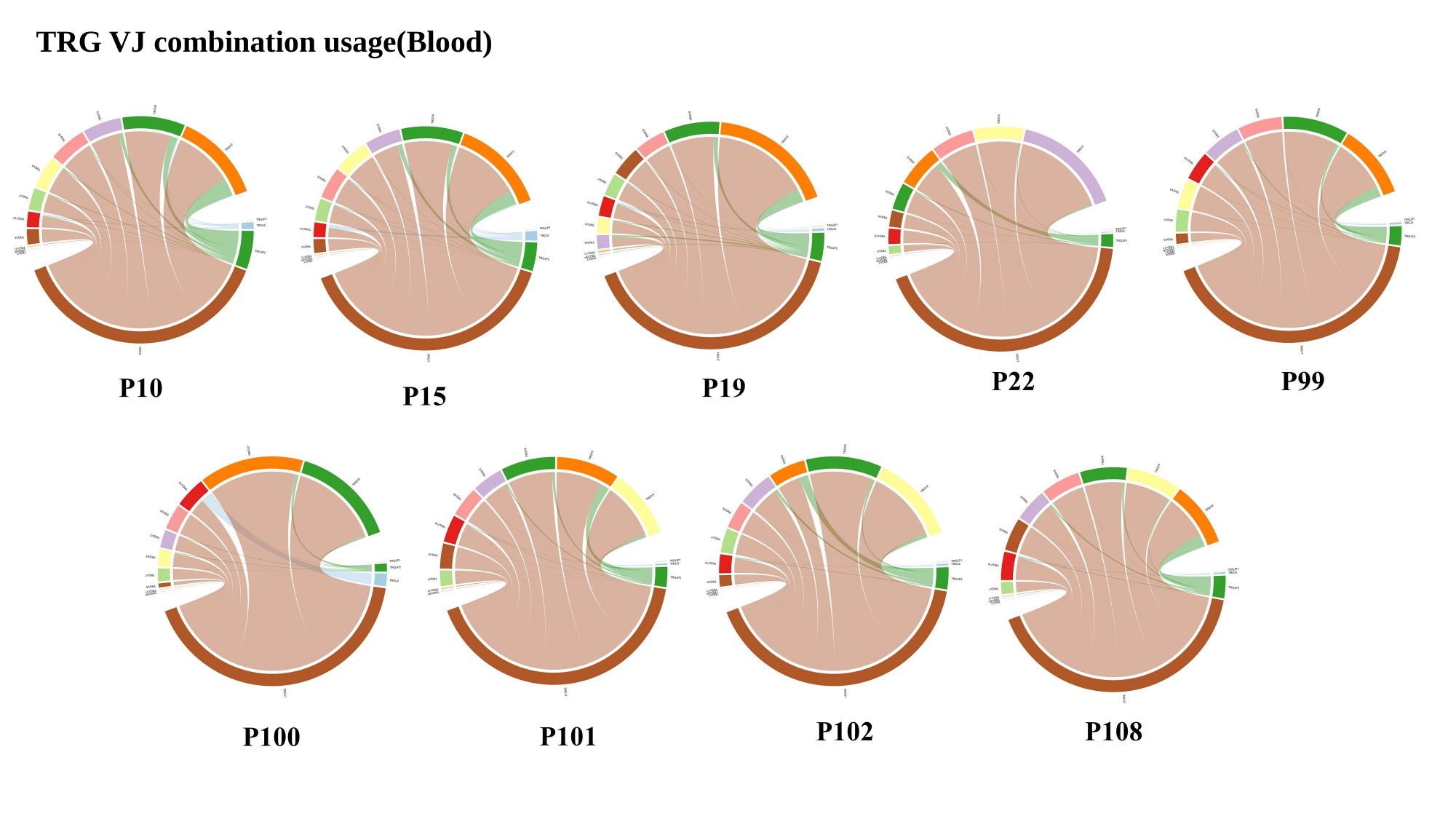

TRG VJ combination usage(Blood)

## Slide 5
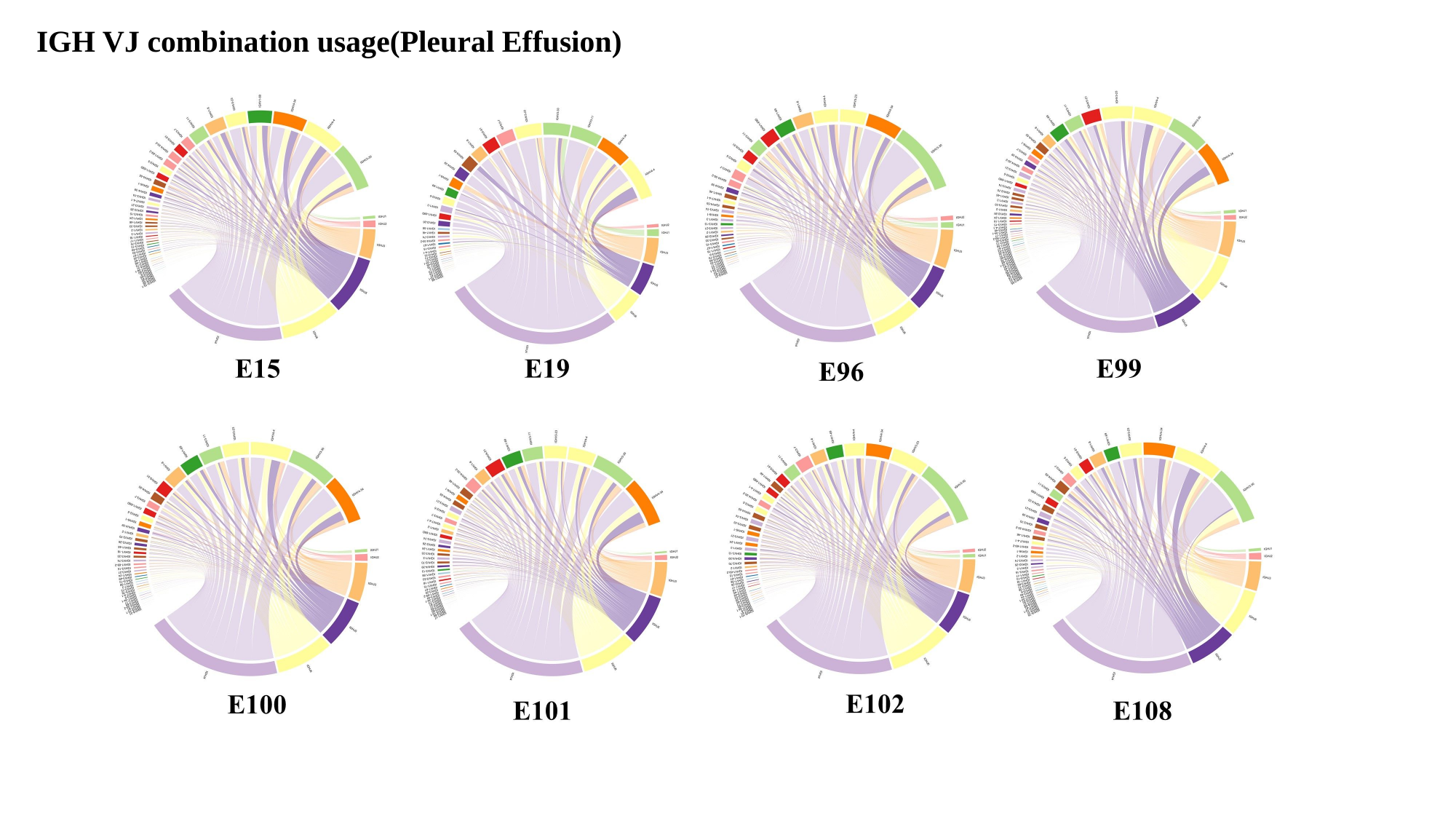

IGH VJ combination usage(Pleural Effusion)

## Slide 6
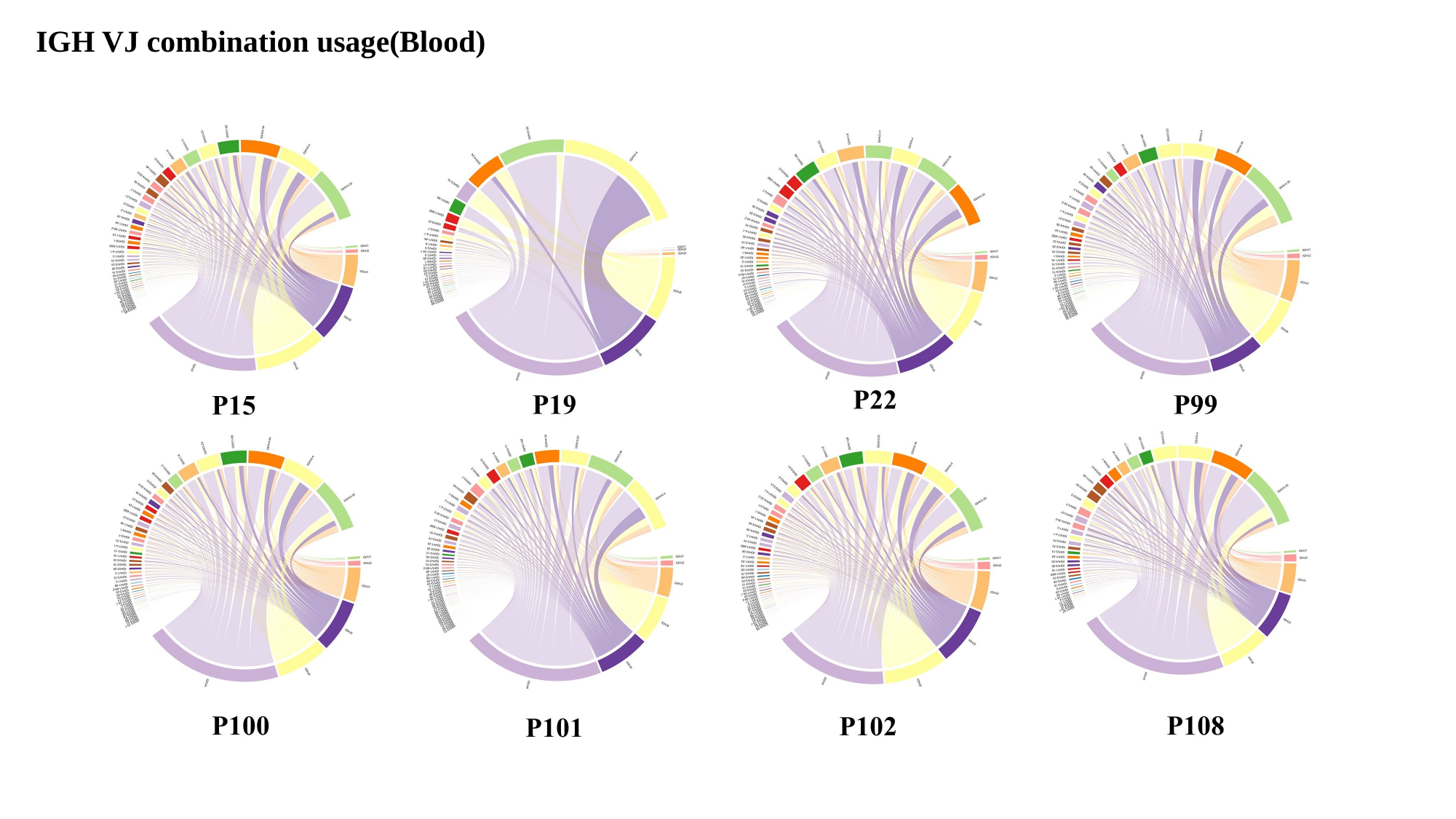

IGH VJ combination usage(Blood)
